# The chromatin remodeler Ino80 mediates RNAPII pausing site determination

**DOI:** 10.1186/s13059-021-02500-1

**Published:** 2021-10-18

**Authors:** Youngseo Cheon, Sungwook Han, Taemook Kim, Daehee Hwang, Daeyoup Lee

**Affiliations:** 1 Department of Biological Sciences, Korea Advanced Institute of Science and Technology, Daejeon, 34141 South Korea; 2 School of Biological Sciences, Seoul National University, Seoul, 08826 South Korea

**Keywords:** The chromatin remodeler Ino80p, Promoter-proximal RNAPII pausing, PRO-seq, Alternative pausing site, + 1 nucleosome, AID system

## Abstract

**Background:**

Promoter-proximal pausing of RNA polymerase II (RNAPII) is a critical step for the precise regulation of gene expression. Despite the apparent close relationship between promoter-proximal pausing and nucleosome, the role of chromatin remodeler governing this step has mainly remained elusive.

**Results:**

Here, we report highly confined RNAPII enrichments downstream of the transcriptional start site in *Saccharomyces cerevisiae* using PRO-seq experiments. This non-uniform distribution of RNAPII exhibits both similar and different characteristics with promoter-proximal pausing in *Schizosaccharomyces pombe* and metazoans. Interestingly, we find that Ino80p knockdown causes a significant upstream transition of promoter-proximal RNAPII for a subset of genes, relocating RNAPII from the main pausing site to the alternative pausing site. The proper positioning of RNAPII is largely dependent on nucleosome context. We reveal that the alternative pausing site is closely associated with the + 1 nucleosome, and nucleosome architecture around the main pausing site of these genes is highly phased. In addition, Ino80p knockdown results in an increase in fuzziness and a decrease in stability of the + 1 nucleosome. Furthermore, the loss of INO80 also leads to the shift of promoter-proximal RNAPII toward the alternative pausing site in mouse embryonic stem cells.

**Conclusions:**

Based on our collective results, we hypothesize that the highly conserved chromatin remodeler Ino80p is essential in establishing intact RNAPII pausing during early transcription elongation in various organisms, from budding yeast to mouse.

**Supplementary Information:**

The online version contains supplementary material available at 10.1186/s13059-021-02500-1.

## Introduction

Emerging evidence indicates that promoter-proximal pausing is a decisive step in transcription that supports the precise control of gene expression in metazoans [1]. In the late 1980s and the early 1990s, several key studies in the Lis group provided insights into a post-initiation block of RNAPII at the uninduced *Hsp* genes using various in vivo analyses [2–5]. These studies revealed that the transcriptionally engaged RNAPII is highly confined at the 5′ end of the gene with a 20–60-nucleotide nascent RNA, however, apparently blocked without heat induction. Thus, the Lis group referred to these promoter-associated RNAPII in uninduced cells as “paused” [6] and first implied its regulatory role in transcriptional activation.

Early biochemical studies in a purified system identified several critical factors that regulate the establishment and release of paused RNAPII. These studies examined the ability of protein factors to confer the sensitivity to 5,6-dichloro-1-β-D-ribofuranosylbenzimidazole (DRB), which has been known to inhibit the synthesis of full-length transcripts. In this system, the two critical factors, DRB sensitivity inducing factor (DSIF; the heterodimeric SPT4/SPT5 complex) [7] and negative elongation factor (NELF) [8], act together to block early elongation, indicating their direct action on RNAPII to establish the paused elongation complex [9].

Initial genome-wide studies based on the chromatin immunoprecipitation (ChIP) method revealed the widespread occurrence of RNAPII accumulation near the transcription start site (TSS) and implicated its role in poising of gene expression in *D. melanogaster* [10–12] and mammalian cells [13, 14]. Advances in genomic technologies further enabled researchers to track the position of elongation complexes genome-wide with higher resolution by several methods, such as by capturing elongation-competent RNAPII [15–17] or selecting RNAPII-associated RNAs [18, 19]. Particularly, the genome-wide nuclear run-on assay, based on the former method, including global nuclear run-on sequencing (GRO-seq) [15] and precision nuclear run-on sequencing (PRO-seq) [16], is able to detect only active RNAPII with the use of detergent sarkosyl.

In *S. cerevisiae*, it has been traditionally believed that much of the transcription regulation occurs during the recruitment step of RNAPII to a gene promoter [20, 21]. According to this view, NELF homologous are absent in fungi [22, 23]. However, interestingly, a previous study using ChIP-chip observed the retention of RNAPII enrichment in *S. cerevisiae* promoter regions [24]. Furthermore, it revealed that the loss of NELF reduced but did not completely abolish RNAPII pausing [25, 26], suggesting that NELF acts to stabilize pausing rather than initiate it. Consistently, several other studies reported that RNAPII pausing is observed in species that lack NELF homologs, such as *C. elegans* [27] and *S. pombe* [28]. In addition, the capture of nascent transcripts in single-nucleotide resolution by native elongating transcript sequencing (NET-seq) in *S. cerevisiae* examined that well-expressed genes exhibit a modest accumulation of read density downstream of TSS [18], resulted in a non-uniform distribution of transcription elongation across genes reminiscent of promoter-proximal RNAPII pausing. Although another previous study using PRO-seq failed to observe this non-uniform distribution of elongating RNAPII, the deletion of Spt4p resulted in a significant increase in the signals immediately downstream of TSS [28], implying the presence of regulation within the promoter-proximal regions of genes in *S. cerevisiae*.

Nucleosome poses a strong barrier for RNAPII passage at various stages of transcription, and cells benefit from employing highly conserved chromatin remodelers to overcome these physical barriers [29]. Biochemical studies demonstrated that RNAPII stalls at the major histone-DNA interaction sites of nucleosome [30], and this could be partially relieved by elongation factors in vitro [31]. Several previous studies also showed that pausing occurs in close proximity to nucleosomes in vivo [18, 28, 32, 33]. A genome-wide study targeting mouse CHD1 revealed that an ATPase inactive form results in a particular increase of RNAPII within the promoter regions [34], implying that chromatin remodeling could affect the promoter escape and subsequent pause-release of RNAPII. A more recent investigation reported the reduced NET-seq signal within promoter-proximal regions and the increase in NET-seq signal at downstream regions in Spt6p depletion (*spt6-1004*). It was accompanied by a striking defect in nucleosome architecture [35], further suggesting the link between early elongating RNAPII and chromatin remodeling. Altogether, despite the close relationship between early transcription elongation and nucleosome, the role of chromatin remodeling in tuning promoter-proximal RNAPII pausing has largely remained elusive.

The chromatin remodeler, Ino80p, has been shown to play a key role in the regulation of RNAPII at transcribed genes through its remodeling activity [36]. Ino80p is thought to exchange the highly conserved histone variant H2A.Z^Htz1^ for H2A [37, 38]. Subsequently, it has been observed that H3K56 acetylation could accelerate dimer exchange reaction by the Ino80 complex in *S. cerevisiae* [39, 40]. However, another group argued that they could not examine the function of the Ino80 complex in driving dimer exchange in *S. cerevisiae* [41, 42]. Apart from its function in dimer exchange, the initially identified nucleosome remodeling activity of Ino80p was nucleosome spacing that usually involves sliding histone octamers along the genomic DNA sequence. The purified Ino80 complex mobilized canonical nucleosome from a lateral to a central position in an ATP-dependent manner [43, 44]. Several studies further examined that Ino80p has the inherent capability of nucleosome spacing and assembly and that it helps organize the intact nucleosome architecture around the promoter by positioning the + 1 nucleosome [45–47] or at specific regions such as centromere [48]. In addition, the Ino80 complex is largely enriched at TSS of genes in yeast and mammals [37, 48–50], and it has been suggested to physically interact with elongating RNAPII, whose serine 2 residue at the C-terminal domain is phosphorylated [51]. Nevertheless, the detailed mechanism through which Ino80p regulates early transcription elongation has mainly remained elusive.

Here, we reveal a non-uniform distribution of elongating RNAPII in *S. cerevisiae* using PRO-seq experiment that detects only elongation-competent RNAPII but not inactive RNAPII such as arrested or unstable form [1]. We examine that these highly confined enrichments of early elongating RNAPII at the 5′ end of genes show both similar and different features with promoter-proximal pausing in *S. pombe* and metazoans. Using the auxin-inducible degron (AID) system [52, 53], we find that Ino80p plays a crucial function in determining the position of RNAPII pausing in *S. cerevisiae*. Genes, whose pausing sites are regulated by Ino80p, exhibit RNAPII pausing at the alternative pausing site even in the physiological condition, and Ino80p is critical in facilitating the utilization of the main pausing site in a nucleosome context-dependent manner. Furthermore, we observe the shift of RNAPII pausing toward the alternative pausing site upon INO80 knockdown also in mESCs, suggesting the essential role of Ino80p in regulating RNAPII pausing in various organisms from budding yeast to mouse.

## Results

### Promoter-proximal pausing-like distributions could be observed in *S. cerevisiae*

**Fig. 1.**
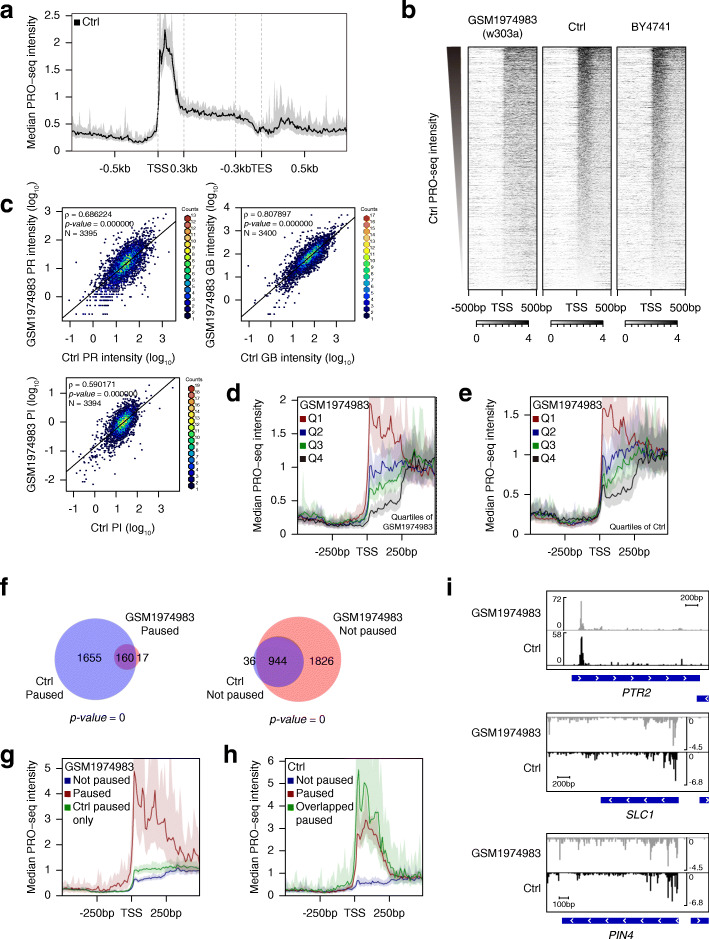
PRO-seq reveals a non-uniform distribution at TSS genome-wide in *S. cerevisiae*. The gene set used in the previous study [28] was used (*N* = 3403) for analysis. **a** Scaled composite profile shows the median intensity of our Ctrl PRO-seq data generated in Ino80p-AID cells cultured without auxin treatment. Regions between 300 bp downstream from TSS and 300 bp upstream of TES were scaled to 60 bins. Outside of these regions were reflected as a 10-bp bin. **b** Heatmaps display previously published *wt* (w303a) PRO-seq data (GSM1974983) and our Ctrl PRO-seq data or *wt* (BY4741) PRO-seq data around TSS. Genes were sorted by the PR intensity of our Ctrl data. Signals reflect the 10-bp bin. **c** Scatterplots represent the correlation of PR and GB intensity and PI between the indicated data. **d**, **e** Average profiles show the median intensity of GSM1974983. Genes were grouped into quartiles based on PI of GSM1974983 (**d**) or Ctrl (**e**) data, where Q1 represents the highest PI. **f** Venn diagram indicates the overlap between paused and not paused genes defined based on GSM1974983 or Ctrl data. *P* value was calculated using the hypergeometric distribution. **g**, **h** Average profiles display the paused and not paused genes defined based on GSM1974983 (**g**; 177 paused genes and 2770 not paused genes) or Ctrl (**h**; 1815 paused genes and 980 not paused genes) data. The green line in the GSM1974983 plot (**g**) indicates 1655 genes defined as paused genes in only Ctrl data. The green line in the Ctrl plot (**h**) represents 160 genes defined as paused genes in both data. **i** Genome browser view of PRO-seq signal for representative genes among overlapped paused genes in both data. PRO-seq data were generated using combined biological replicates

To investigate the genome-wide distribution of elongation-competent RNAPII in *S. cerevisiae*, we performed PRO-seq [17, 28] with 2-Biotin run-on (biotin-11-CTP and UTP) with *S. pombe* spike-in control. We used Ino80p-AID cells [52] cultured without auxin treatment, which is hereafter referred to as a control (Ctrl) condition. We measured the reproducibility between two biological replicates as Spearman’s *ρ* (Additional file 1: Table S1, SC_Ino80p-AID_Ctrl; the table included the summary of PRO-seq reads and the reproducibility of all the biological replicates used in this study). PRO-seq revealed that transcription elongation was non-uniformly distributed: It was concentrated near TSS, with a pattern resembling that associated with promoter-proximal pausing in metazoans (Fig. 1a). This was surprising because a previous study using PRO-seq in *S. cerevisiae* had captured a relatively uniform distribution of RNAPII across genes [28].

To examine the plausible reasons for this discrepancy, we analyzed the reported PRO-seq data set [28] and compared it to our Ctrl data. We first sorted the gene set used in the previous study based on the promoter-proximal intensity in our Ctrl data. Since we compared two independently generated data, the experimental reads were normalized to reads per million mapped reads (RPM) without considering spike-in counts. Interestingly, the heatmap around TSS of the reported *wt* (w303a) data (GSM1974983) exhibited a generally similar order of signals to those of our data (Fig. 1b). We also calculated PRO-seq intensity within the promoter-proximal (PR; TSS to TSS + 250 bp) and the gene body (GB; TSS + 250 bp to transcription end site (TES)) regions, and pausing index (PI; a ratio of PR density to GB density) for each data, and demonstrated a positive correlation (as Spearman’s *ρ*) between two data (Fig. 1c). PRO-seq density was calculated as the number of sense strand PRO-seq reads per mappable bases within the indicated region as described in the previous study [15, 28] (See “Methods”). To examine the possibility that this non-uniform PRO-seq distribution is a strain background-specific result, we also performed PRO-seq experiments in *wt* (BY4741) cells and found a strikingly similar pattern around TSS with Ctrl data (Fig. 1b).

In order to demonstrate whether the previous data showed any indication of pausing, we binned genes in quartiles based on PI. We then compared the average profile of each quartile. Interestingly, we found that the gene group with the highest PI (Q1) showed the strikingly accumulated signal downstream of TSS, resembling the distribution of RNAPII pausing (Fig. 1d). As consistent with the positive value of Spearman’s *ρ* between PI of two data, we observed that the previous data was also generally well sorted in order of PI of our data (Fig. 1e). Next, we classified genes as being paused or not paused based on the significance of PI that was calculated by Fisher’s exact test with Bonferroni’s correction as in the previous study [28] (See “Methods”). We were able to classify 177 paused genes (Fig. 1f, left) that showed the accumulated PRO-seq signals within PR regions in the previous data (Fig. 1g, the red line). We also found that most of them were overlapped with the paused genes classified based on our Ctrl data (Fig. 1f, left). Although 1655 genes were not classified as paused in the previous data, they exhibited higher PR intensity than not paused genes (Fig. 1g; see the blue and green line). Moreover, 160 genes classified as paused in both data displayed a higher PRO-seq intensity than the total paused genes in our Ctrl data (Fig. 1h; see the red and green line). These results indicate that first, the previous data clearly exhibited paused RNAPII at least for a subset of genes, and second, the PR intensity in the previous data was mainly enriched at genes with relatively high PR intensity in our data. For the reasons above, we speculate that RNAPII engaged in PR regions might be more sensitively detected in our data than in the previous data. In addition, we found that the mapped reads in the previous data [28] were lower than half of those in our data which might also affect the overall sequencing depth. One difference between two data was that our PRO-seq was performed using 2-Biotin run-on instead of 4-Biotin run-on [28], leading to a decrease in the resolution. However, based on two points that first, 2-Biotin run-on provides reasonable resolution [17], and that second, GRO-seq, the lower resolution approach of PRO-seq, agrees well with PRO-seq in general [16], the difference in PRO-seq distribution near the 5′ end of genes was not likely attributed to a decrease in resolution caused by 2-Biotin run-on. Accordingly, we observed strikingly similar patterns of two PRO-seq data at the representative overlapped paused genes (Fig. 1i).

As the previous study reported the PRO-seq pattern in *spt4*Δ as well [28], we next performed PRO-seq experiments in the *spt4*Δ cells to examine whether our PRO-seq experiments reproduce the previous data. Analysis of PRO-seq intensity within PR and GB regions also demonstrated a positive correlation between the previous PRO-seq data (GSM1974984) and our PRO-seq data generated in *spt4*Δ (Additional file 1: Fig. S1a, b). Consistent with the previous report [28], we found that *spt4*Δ caused a significant increase of PRO-seq intensity at the 5′ end of the gene, resulting in the increasing PI (Additional file 1: Fig. S1c, d). This result additionally indicated the reliable validity and reproducibility of our PRO-seq experiments.

To further substantiate the integrity of our PRO-seq data, we also performed PRO-seq in *S. pombe* and mESCs and compared them to the published data sets [28, 54]. For *S. pombe* data, the gene set used in the previous study (*N* = 3214) [28] was used for analysis. Our *wt* (ED665) PRO-seq data showed a highly correlated pattern with the previously reported data generated in *wt* (972) cells (GSM1974985) [28] within both PR and GB regions (Additional file 1: Fig. S2a, b). We also observed that PIs of two different data were highly correlated (Additional file 1: Fig. S2b, c, and d). As in the *S. cerevisiae* data, we classified more paused genes in our data than the previous data in *S. pombe* (Additional file 1: Fig. S2e, left). Consistently, although 993 genes were not classified as being paused in the previous data, they exhibited higher PR intensity than not paused genes (Additional file 1: Fig. S2f; see the blue and green line). In addition, 587 genes classified as being paused in both data displayed a higher PRO-seq intensity than the total paused genes in our data (Additional file 1: Fig. S2g; see the red and green line). These further supported the speculation that our PRO-seq data seemed to more sensitively detect PR RNAPII than the previous data. We also found a strong positive correlation between the previously reported PRO-seq data [54] and our data generated in mESCs when analyzing total protein-coding genes (*N* = 38,984) (Additional file 1: Fig. S3). Based on these collective results, we demonstrate that our PRO-seq data was highly correlated with the previously published data in all *S. cerevisiae*, *S. pombe*, and mESCs. Thus, we conclude that the non-uniform distribution of PRO-seq signal within PR regions in *S. cerevisiae* shown in our data is not attributed to experimental or analytical bias.

To further examine the genome-wide significance of these apparent pausing-like features in *S. cerevisiae*, we focused on analyzing distribution in Ctrl PRO-seq data at RNAPII-transcribed protein-coding genes. We also performed precision nuclear run-on 5′ cap sequencing (PRO-cap) with 2-Biotin run-on (biotin-11-CTP and UTP) [17] in the Ctrl condition to define the transcription initiation sites more precisely (Additional file 1: Fig. S4a). We chose the single base pair with the most PRO-cap reads within 250 bp upstream and downstream of the annotated TSS. We found the specific sequence preference markedly similar to that detected by the previous studies (Additional file 1: Fig. S4b) [28, 35]. Thus, we herein refer to the newly defined observed TSS as a “TSS” unless otherwise noted.

We first tested whether the Ctrl PRO-seq signal around TSS was consistent with the previously published *wt* Rpb3p NET-seq [18] and ChIP-exo [55], independent assays for detecting elongation complexes and paused RNAPII [1]. We generated the heatmaps centered on TSS that were sorted by PR intensity of Ctrl PRO-seq data using total filtered protein-coding genes (*N* = 5697; see “Methods”). Both NET-seq and ChIP-exo data exhibited the enriched signal near TSS similar to PRO-seq data (Additional file 1: Fig. S5a). Moreover, scatter plots showed a strong positive correlation between NET-seq and PRO-seq data (Additional file 1: Fig. S5b) and a positive correlation between ChIP-exo and PRO-seq data (Additional file 1: Fig. S5c) in both PR and GB regions. The similarity between PRO-seq and NET-seq was much stronger than previously suggested [28]. We also demonstrated the positive correlation between NET-seq and ChIP-exo data (Additional file 1: Fig. S5d), indicating analogous distribution patterns of all PRO-seq, NET-seq and ChIP-exo data across genes in *S. cerevisiae*. In addition, we analyzed the previously reported BioGro data, another nuclear run-on technique [56]. Interestingly, we found a positive correlation between BioGro data and PRO-seq data in PR and GB regions (Additional file 1: Fig. S5e). We speculate that a relatively low but moderately positive correlation value (*ρ* = 0.526) of PR intensity might be because BioGro data was generated based on the tiling array, which could not show the high resolution as much as PRO-seq.

**Fig. 2:**
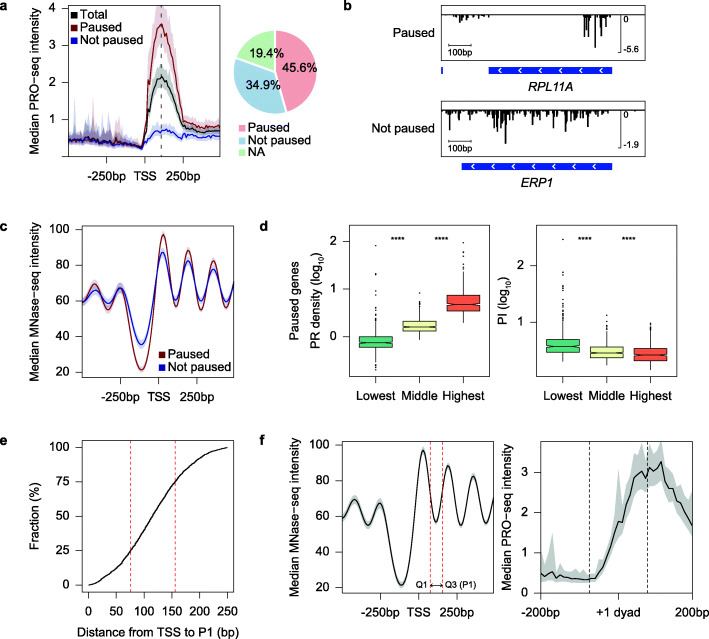
Features of the non-uniform distribution of transcription elongation in *S. cerevisiae*. **a** Average profile shows median PRO-seq intensity around TSS (left). In total, 2599 paused and 1990 not paused genes were classified among 5697 total filtered protein-coding genes (right). **b** Genome browser view of PRO-seq signals for representative paused (top) or not paused genes (bottom). **c** Average profile indicates median MNase-seq (GSM3304635) intensity centered on TSS of paused and not paused genes. **d** Boxplots exhibit the relationship of PRO-seq PR density (left) and PI (right) to the bottom (0–20%), middle (40–60%), and top (80–100%) pentiles of gene activity. Only paused genes were considered. Asterisks represent statistically significant differences based on Mann-Whitney U test. **e** Cumulative curve analyzes the distance from TSS to P1 at paused genes. The red dotted lines represent the 25th (76 bp) and 75th (156 bp) percentiles. **f** Average profiles show median MNase-seq intensity (GSM3304635) at TSS (left) and median PRO-seq intensity at the + 1 dyad (right). The + 1 dyad was defined by the improved nucleosome-positioning algorithm (iNPS) using existing MNase-seq data (GSM3304635) [57]. The two dotted red lines represent the 25th (Q1; 76 bp) and 75th (Q3; 156 bp) percentiles of P1 (left), and the two dotted black lines represent the expected position of the + 1 nucleosome (right; 75 bp upstream and downstream of + 1 dyad). All PRO-seq data represent the Ctrl data that were generated in auxin-untreated Ino80p-AID cells and combined biological replicates were used. For average profiles, medians reflect the 10-bp bin

We next classified protein-coding genes as being paused or not paused as described above. We identified 2599 (45.6%) high-confidence paused and 1990 (34.9%) not paused genes among 5697 filtered protein-coding genes (Fig. 2a, b; NA represents the genes that were classified as neither). The prevalence of pausing in *S. cerevisiae* was thus higher than that previously observed in *S. pombe* (28%) [28] and human (41%) [15] but lower than that observed in *D. melanogaster* (63%) [25]. Furthermore, as consistent with our gene classification results, both NET-seq and ChIP-exo data exhibited much higher enrichment at TSS of paused genes than those not paused genes (Additional file 1: Fig. S5f, g). The difference in the peak position shown in the average profiles of three different experimental data, PRO-seq, NET-seq, and ChIP-exo, might reflect a distinct method of each technique.

Next, we sought to identify the features of the PRO-seq pattern in *S. cerevisiae*. First, we generated heatmaps of PRO-seq, PRO-cap, and existing data of MNase-seq [52] and ChIP-seq against TBP and phospho-Ser5 of RNAPII C-terminal domain (pSer5) [58]. PRO-seq intensity within PR regions generally correlated with the enrichment of TBP and RNAPII pSer5 for both paused and not paused genes (Additional file 1: Fig. S6a), which implied that TBP and pSer5 intensity does not necessarily point to RNAPII pausing. In addition, the heatmaps showed that the higher the PRO-seq signal, the lower the nucleosome occupancy and the wider the nucleosome-free region (NFR) (Additional file 1: Fig. S6a) as consistent with an earlier report based on NET-seq signal [59]. We also revealed that nucleosome distribution around TSS of paused genes displayed a more highly phased pattern than those of not paused genes (Fig. 2c), in agreement with the previous study in *S. pombe* [28].

At protein-coding genes, gene activity, which was determined by PRO-seq GB density [15], generally correlated with PRO-seq PR density (Fig. 2d, left), whereas it was inversely correlated with PI (Fig. 2d, right). However, we did not find these correlations when considering all genes. PI of a group of genes with the lowest gene activity was not significantly higher than those with the middle gene activity (Additional file 1: Fig. S6b, right). In contrast, a previous study in human lung fibroblasts reported an inverse correlation between gene activity and PI regardless of whether considering only paused genes or all genes [15]. This might be because gene activity of not paused genes in *S. cerevisiae* was lower than generally in humans. Indeed, we found that about half were not paused genes in the group of genes with the lowest gene activity, whereas about one-third or a quarter was not paused genes in the groups of middle and highest gene activity. Based on our collective results, we propose that RNAPII distributions at the 5′ end of genes in *S. cerevisiae* are an aspect of early transcription elongation that showed similar characteristics with PR pausing in *S. pombe* and metazoans.

### Pausing-like feature in budding yeast is broader and more distal than that in metazoans

Despite the observed similarities described above, we also examined a striking difference in PRO-seq distributions of *S. cerevisiae* compared to metazoans*.* The peak of average PRO-seq enrichment within PR regions was located ~ 100 bp downstream of TSS (Fig. 2a, left). This location was much farther downstream than the peak of average GRO-seq and PRO-seq enrichment in metazoans, which showed the read peaks at ~ 50 bp downstream of TSS [15, 16]. To analyze the PRO-seq distribution in *S. cerevisiae* more precisely, we tried to define the main pausing site for each gene. First, we smoothed PRO-seq reads within PR regions with a Savitzky-Golay smoothing method [60] to increase the accuracy and precision without distorting the PRO-seq signal. We next designated peak showing the highest mean reads for two replicates in Ctrl samples as P1 (See “Methods”), similar to the identification of the 1st pausing site in a recent study [33]. The cumulative curve demonstrated that the 25th and 75th percentiles of the distance from TSS to P1 were 76 and 156 bp, respectively (Fig. 2e).

To determine the association between PR RNAPII distribution and + 1 nucleosome, we generated the average profile of existing MNase-seq [52] around TSS (Fig. 2f, left) and our Ctrl PRO-seq data around + 1 nucleosome dyad (Fig. 2f, right). Interestingly, the majority of P1 was found to be located downstream of the + 1 nucleosome. The 25th and 75th percentiles of the distance from TSS to P1 are represented as two dotted red lines. This result is consistent with the previous report using NET-seq, which showed the peak of mean pause density downstream of the + 1 dyad [18]. In contrast, RNAPII is generally paused upstream of the + 1 dyad in metazoans [16, 32, 33].

### Ino80p is critical for the proper positioning of RNAPII at genes with the alternative pausing site

The highly conserved chromatin remodeler Ino80p is expected to have a putative role in regulating PR RNAPII distributions given that it has been not only shown to localize around the 5′ end of genes [37] and but also found to be critical in establishing the nucleosome architecture around the promoter [45–47]. Thus, we performed PRO-seq to investigate the role of Ino80p in nascent transcription across PR regions at nearly single-nucleotide resolution. We employed the AID system [52, 53], with the goal of observing an immediate effect of Ino80p knockdown in transcription elongation. Briefly, Ino80p-AID cells were grown to the mid-log phase in yeast peptone dextrose (YPD) containing ethanol (Ctrl; the same condition as described above). Ethanol was washed from the media, and the cells were incubated with auxin (0.5 mM) for 3 h (KD). Auxin was washed from the media, and cells were incubated in an auxin-free medium for an additional 3 h (Rescue) (Additional file 1: Fig. S7a). Western blot analysis confirmed the conditional depletion and recovery of Ino80p in an AID-tag-dependent manner after the 3 h incubations with or without auxin (Additional file 1: Fig. S7b). We also noted the strikingly similar PRO-seq pattern between the data generated using cells incubated with YPD containing ethanol and those with YPD only (Fig. 1b; Ctrl and BY4741). Both PR and GB intensity of two data correlated well (*ρ* = 0.9393586 for PR and *ρ* = 0.9606225 for GB). It confirmed that the addition of a small volume of ethanol to YPD did not affect elongating RNAPII distribution across genes.

**Fig. 3:**
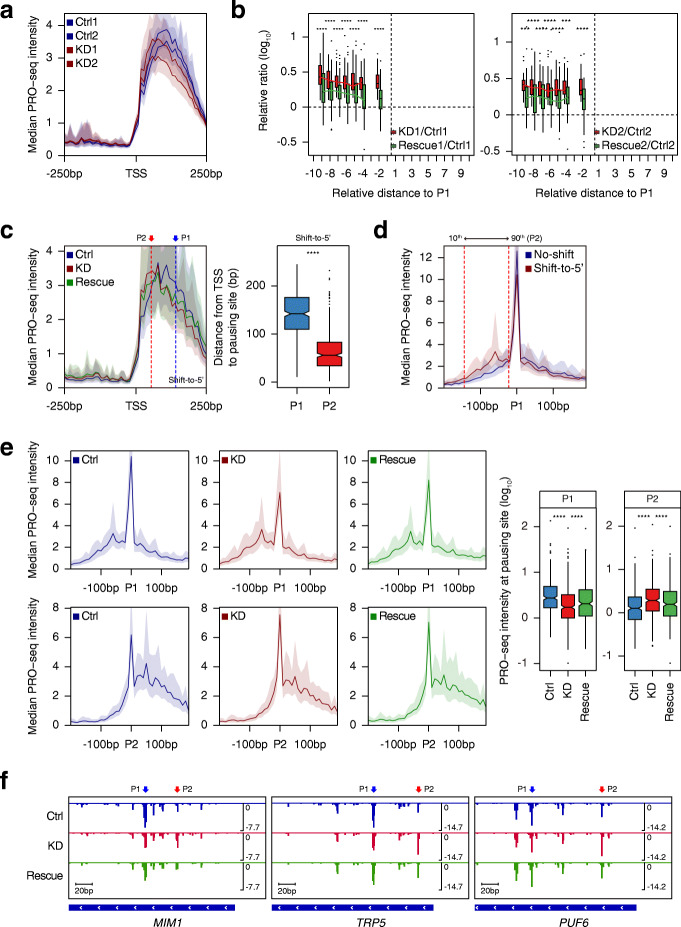
Ino80p knockdown causes the transition of RNAPII pausing at genes with the alternative pausing site. **a** Average profile indicates median PRO-seq intensity in Ino80p-AID cells under Ctrl and KD conditions for paused genes. **b** Boxplots represent the relative read ratio (log_10_) at the significantly increasing peaks (red indicates the ratio of KD versus Ctrl and green indicates the ratio of Rescue versus Ctrl) at each replicate. To analyze the relative distance to P1 from each peak (*x*-axis), we first calculated the distance either from TSS to P1 or P1 to TSS + 250 bp, and divided each into 10 bins, and mapped the peak to the corresponding bins. Former and later bins were referred to as − 10 to − 1 and 1 to 10 depending on the distance from P1, respectively. To display the significantly enriched bins, bins with less than 5% of the total increasing peaks were excluded; in the end, 386 peaks for replicate 1 and 383 peaks for replicate 2 out of 618 peaks at 467 genes were used. **c** Average profiles exhibit median PRO-seq intensity at TSS of shift-to-5′ genes (left). The arrows and dotted lines represent the median of P1 (blue, 142 bp) and P2 (red, 56 bp). Boxplot shows the distance from TSS to the indicated pausing sites (right). **d** Average profiles represent median PRO-seq intensity for no-shift and shift-to-5′ genes in Ctrl data. The two dotted lines represent the 10th (− 22 bp) and 90th (− 144.8 bp) percentiles of P2 relative to P1 for shift-to-5′ genes. **e** Average profiles display median PRO-seq intensity (left), and boxplots depict the smoothed PRO-seq intensity (right) at the indicated pausing sites of shift-to-5′ genes. **f** Genome browser view of PRO-seq signal for representative shift-to-5′ genes. All data except for (**a**) were generated using combined biological replicates. For average profiles, medians reflect the 10-bp bin. Asterisks represent statistically significant differences based on Wilcoxon signed rank test

Interestingly, we found that PRO-seq experiments under Ino80p knockdown (Ino80p-KD) caused an upstream skewed pattern of the peak within PR regions (Fig. 3a). The experimental reads were normalized to the relative number of spike-in counts, and these normalized reads were counted in RPM considering the sequencing depth of experimental reads. To demonstrate that this upstream skew was due to the increase of the PRO-seq signal at the upstream of P1, we first identified increasing peaks within PR regions upon Ino80p-KD. Briefly, we defined all the consensus peaks detected in the data set and calculated each peak’s read ratios to P1. We then measured the significance of changes in these read ratios upon Ino80p-KD based on an empirical statistical test considering the variance of each replicate as previously reported [61] (See “Methods”). We finally chose significantly increasing 618 peaks for 467 genes upon Ino80p-KD. To examine whether most of the increasing peaks were located upstream of P1, we calculated the distance from TSS to P1 and from P1 to TSS + 250 bp. We then divided each into 10 bins. Former and later bins were referred to as − 10 to − 1 and 1 to 10 depending on the distance from P1, respectively. The increasing peaks were mapped to the designated bins. In order to display significantly enriched bins, bins with less than 5% of the total increasing peaks were excluded. As expected, we found that most of the increasing peaks were located upstream of P1 at each replicate (Fig. 3b). Furthermore, increasing read ratios upon Ino80p-KD (Fig. 3b, the red box) was significantly decreased under auxin withdrawal (Fig. 3b, the green box), indicating the Ino80p-dependent regulation of these peaks.

We next classified genes based on the relative position of increasing peak to P1 and referred to 353 genes with the increasing upstream peaks as “shift-to-5′ genes” and 150 genes with the increasing downstream peaks as “shift-to-3′ genes”. For both gene sets, only some of the genes exhibited multiple increasing peaks, so that we selected increasing peak with the lowest *p* value for those genes in an effort to focus on the most significantly changed peak upon Ino80p-KD (77 genes out of 353 shift-to-5′ genes and 12 genes out of 150 no-shift genes showed multiple increasing peaks). We designated these selected increasing peaks in Ino80p-KD as P2. To compare the significance of increase at P2 for shift-to-5′ and shift-to-3′ genes, we displayed heatmaps of the PRO-seq log_2_ fold change around P1 (Additional file 1: Fig. S8a). Consistent with the boxplot data (Fig. 3b), the increasing upstream P2 was robust and more frequent than increasing downstream P2. Next, we additionally selected genes with non-increasing peaks (Additional file 1: Fig. S8b; we observed almost no increase in read ratios upon Ino80p-KD and non-significant changes under subsequent restoration) and referred to 350 genes as “no-shift genes”. Since P1 and P2, and even non-increasing peaks, were selected considering the variance of replicates, we used the combined biological replicates to display the data in further analysis. As expected, we observed a significant increase in PRO-seq signals at P2 under Ino80p-KD for shift-to-5′ genes, and that subsequent rescue of Ino80p caused a tendency to counteract this increase (Fig. 3c). In contrast, there was no significant increase in PRO-seq signal for no-shift genes and even for shift-to-3′ genes (Additional file 1: Fig. S8c). Thus, we conclude that the role of Ino80p is mainly associated with the upstream P2, thereby focusing on our further analysis for shift-to-5′ genes. Overall, these results indicate that Ino80p plays a previously unrecognized function in the proper localization of RNAPII in the early elongation stage for a subset of genes.

To investigate the general features of genes whose PR RNAPII distributions were dependent on Ino80p, we compared the PRO-seq pattern at P1 under the Ctrl condition for no-shift and shift-to-5′ genes. Surprisingly, we observed a distinct feature between the two gene groups. No-shift genes displayed a sharp and distinct peak, whereas shift-to-5′ genes exhibited a lower enrichment at P1 accompanied by an accumulation of PRO-seq signal at the upstream of P1 (Fig. 3d). We further investigated the use of P1 by RNAPII for each gene set by quantifying the ratio of P1 reads to total reads of peaks within PR regions (i.e., retained ratio at P1). Although the total reads were not significantly different between the two gene groups, retained ratio at P1 for no-shift genes was significantly higher than shift-to-5′ genes (Additional file 1: Fig. S8d). These results indicate more use of the alternative pausing site by RNAPII in shift-to-5′ genes than in no-shift genes. We also found that the majority of P2 was located where the upstream accumulation occurs in shift-to-5′ genes (Fig. 3d; the 10th and 90th percentiles of the relative position of P2 to P1 for shift-to-5′ genes are represented by the dotted lines). This overlapped location indicates that Ino80p-KD relocates RNAPII to, not random, but particular positions where pausing weakly occurs in the physiological condition. Furthermore, we observed that the PRO-seq signal at P1 of shift-to-5′ genes was significantly decreased upon Ino80p-KD, while the accumulated signal upstream of P1 was maintained (Fig. 3e, average profiles, top). When aligned at P2, the PRO-seq signal was even significantly increased upon Ino80p-KD (Fig. 3e, average profiles, bottom), and both changes tended to reset upon subsequent Ino80p rescue. We additionally noted that the PRO-seq signal at P2 in Ctrl condition was above the basal level, further indicating that RNAPII could pause at P2 in the physiological condition and that RNAPII at shift-to-5′ genes use P2 as the alternative pausing site. Genome browser view of PRO-seq distribution at representative shift-to-5′ genes additionally showed that the transition of RNAPII upon Ino80p-KD was not biased by the average profile (Fig. 3f). Interestingly, the sequence preference at P1 and P2 of shift-to-5′ genes generated by WebLogo [62] exhibited a marked similarity (Additional file 1: Fig. S8e, top), further supporting the usage of P2 as the alternative pausing site. In contrast, we found no sequence preference at the regions that did not arise from the pausing site, which was the middle of GB regions (Additional file 1: Fig. S8e, bottom). It demonstrates that the specific sequence preference was uniquely identified at the pausing site and not artifactual bias by the biotin incorporation. Based on our collective results, we conclude that Ino80p plays a role in determining proper pausing site on genes with the alternative pausing site.

We next determined whether the observed transition of RNAPII pausing was due to a defect in TSS usage. The precise transcription initiation sites for shift-to-5′ genes upon Ino80p-KD were identified in the same manner as Ctrl data. A histogram of the distance between TSS in Ctrl and KD data indicated that most of these genes exhibited no differences in transcription initiation sites (Additional file 1: Fig. S8f). This result suggests that the Ino80p-dependent positioning of RNAPII pausing is primarily caused by a defect in transcription elongation rather than a defect in TSS usage.

Given that previous reports indicating a connection between Ino80p and H2A.Z^Htz1^ [37, 38], we questioned whether the transition of RNAPII upon Ino80p-KD is associated with H2A.Z^Htz1^. To test this possibility, we carried out PRO-seq experiments in *htz1*Δ cells. However, the PRO-seq average profile revealed that *htz1*Δ did not result in a skewed pattern of the PR peak for shift-to-5′ genes as upon Ino80p-KD (Additional file 1: Fig. S8g, left). Moreover, H2A.Z^Htz1^ enrichment in the + 1 nucleosome at no-shift and shift-to-5′ genes, which was calculated from an existing MNase-ChIP-seq in *wt* cells [63], showed no significant difference (Additional file 1: Fig. S8g, right). Thus, we conclude that the function of Ino80p in pausing site determination for shift-to-5′ genes is independent of H2A.Z^Htz1^.

### The transition of promoter-proximal RNAPII is closely associated with the + 1 nucleosome

**Fig. 4:**
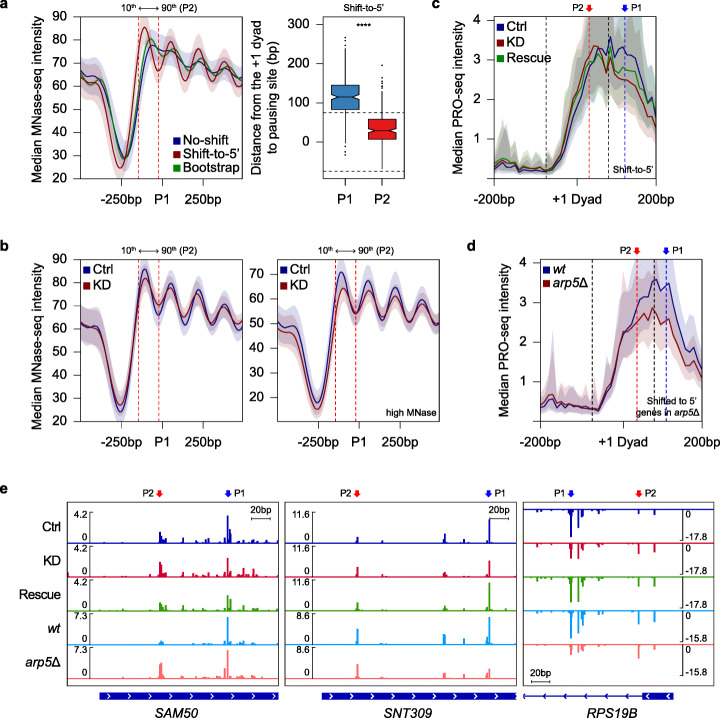
Proper localization of pausing is closely associated with the regulation of + 1 nucleosome by Ino80p. **a** Average profile shows median MNase-seq intensity in the auxin-untreated Ino80p-AID cells (GSM3304635) at P1 of no-shift and shift-to-5′ genes (left). The green line exhibits the median of 1000 bootstrap samples (Each sample containing the same number of genes to the original population and was obtained by resampling allowing replacement). Boxplot represents the distance from the + 1 dyad to the indicated pausing sites for shift-to-5′ genes (right). **b** Average profiles depict median MNase-seq intensity at P1 of shift-to-5′ genes in the auxin-untreated (Ctrl) or -treated (KD) data. GSM3304635 and GSM3304637 were used for the left panel, and GSM3177778 and GSM3177779 were used for the right panel (high MNase). For (**a**) and (**b**), the two dotted lines indicate the 10th (− 22 bp) and 90th (− 145 bp) percentiles of P2 relative to P1. **c**, **d** Average profiles display median PRO-seq intensity for the indicated samples around the + 1 dyad. The two dotted lines in profiles represent the expected position of the + 1 nucleosome (75 bp upstream and downstream of the + 1 dyad). **e** Genome browser view of PRO-seq signals for representative genes whose pausing sites were shifted in the 5′ direction in both Ino80p-KD and *arp5*Δ. All PRO-seq data were generated using combined biological replicates. Only genes with nucleosomes overlapped with H3K4me3 ChIP-seq enrichment (GSM2507874) were used in an effort to exclude false-positive nucleosomes (260 no-shift genes and 251 shift-to-5′ genes upon Ino80p-KD; 398 shifted to 5′ genes in *arp5*Δ). For average profiles, medians reflect the 10-bp bin. Asterisks represent statistically significant differences, as calculated using either Wilcoxon signed rank test or Mann-Whitney *U* test

We questioned whether the transition of RNAPII upon Ino80p-KD is associated with the nucleosome architecture around P1. To address this, we first analyzed the average profile of existing MNase-seq data generated in the auxin-untreated Ino80p-AID cells [52]. To discard false-positive nucleosome positions, we excluded nucleosomes that did not overlap H3K4me3 ChIP-seq enrichment calculated from the existing data [64], as previously reported [33]. Surprisingly, the nucleosome distribution around P1 of shift-to-5′ genes displayed a much better phase than no-shift genes and even bootstrapped estimation (Fig. 4a, left). In addition, at shift-to-5′ genes, + 1 nucleosome tended to be located in much closer proximity to P2 than to P1 (Fig. 4a, right). While the majority of P1 was located downstream of the + 1 nucleosome, the majority of P2 was located between the + 1 dyad and the + 1 nucleosome boundary.

We next examined existing MNase-seq data obtained upon Ino80p-KD [52] and found that nucleosome distribution around P1 of shift-to-5′ genes exhibited a moderate disturbance in nucleosome positioning accompanied by a slight decrease in + 1 nucleosome occupancy (Fig. 4b, left). Such decrease was further emphasized by nucleosome digestion with high MNase from another recent report [58] (Fig. 4b, right). Nevertheless, we did not find a striking difference in Ino80p enrichment, which was calculated from an existing ChEC-seq data [58], between no-shift and shift-to-5′ genes (Additional file 1: Fig S9a).

To more precisely analyze the association between RNAPII pausing and + 1 nucleosome, we investigated the PRO-seq distribution around the + 1 dyad upon Ino80p-KD. We observed significantly reduced PRO-seq signal downstream of the + 1 nucleosome upon Ino80p-KD, and rescue of Ino80p tended to reset this change (Fig. 4c). Such change in PRO-seq distribution upon Ino80p-KD was also found in the profile centered on the + 1 dyad defined based on MNase-seq generated in Ino80p-KD (Additional file 1: Fig. S9b). Consistently, we examined that retained ratio at P1 was significantly decreased upon Ino80p-KD and recovered upon subsequent restoration, whereas those at P2 showed an opposite tendency (Additional file 1: Fig. S9c). These quantifications demonstrate that Ino80p-KD caused a significant increase in the ratio of P2-associated RNAPII to total PR RNAPII. In contrast, we did not find significant changes in PRO-seq distribution around + 1 nucleosome upon Ino80p-KD for no-shift genes (Additional file 1: Fig. S9d; no-shift genes were divided by whether they exhibited P1 outside or inside of the + 1 nucleosome to distinguish the changes in PRO-seq distribution clearly).

To investigate whether the regulation of nucleosome architecture around the pausing site depends on the Ino80 complex, we carried out PRO-seq experiments in *arp5*Δ cells that lack a component essential for the chromatin remodeling activity of the Ino80 complex in *S. cerevisiae* [65–67]. When we analyzed the defined paused genes as described for Ino80p-KD data, we observed that *arp5*Δ also mainly caused a shift of pausing site toward the 5′ direction (Additional file 1: Fig. S9e). In addition, P2 of shifted to 5′ genes in *arp5*Δ is located more closely to + 1 dyad than P1 (Additional file 1: Fig. S9f). Furthermore, we examined that *arp5*Δ led to a particular decrease in PRO-seq signal downstream of + 1 nucleosome (Fig. 4d) accompanied by a significant increase in retained RNAPII at P2 (Additional file 1: Fig. S9g). In contrast, not shifted genes did not show a distinct change of PRO-seq distribution around + 1 dyad in *arp5*Δ (Additional file 1: Fig. S9h). Corroborating this, a Venn diagram analysis revealed a significant overlap (*p* value = 6.95 × 10^−12^) between shift-to-5′ genes upon Ino80p-KD and shifted to 5' genes in *arp5*Δ (Additional file 1: Fig. S9i). Genome browser view images further exhibited a transition of elongating RNAPII from P1 to P2 for representative genes in both Ino80p-KD and *arp5*Δ (Fig. 4e). Collectively, we conclude that the regulation of the + 1 nucleosome by the Ino80 complex is strongly associated with positioning of PR RNAPII.

### The transition of promoter-proximal RNAPII upon the loss of INO80 is also observed in mESCs

Since the Ino80 complex is highly conserved from yeast to human [36], we investigated whether INO80 loss also caused a defect in elongating RNAPII positioning in mESCs. Toward this end, we carried out PRO-seq experiments in mESCs treated with either *siEGFP* or *siINO80* (Additional file 1: Fig. S10a). To identify the position of PR pausing more precisely, we tiled 1 kb around the annotated TSSs in a 50-bp window with a 5-bp shift, as previously reported [15]. We selected a window showing maximum PRO-seq reads and designated the 5′ end of the selected window as the “5′-peak”. The average profile of median PRO-seq intensity in mESCs treated with *siEGFP* revealed almost 2-fold higher PRO-seq intensity at 5′-peak than annotated TSS (Additional file 1: Fig. S10b).

**Fig. 5:**
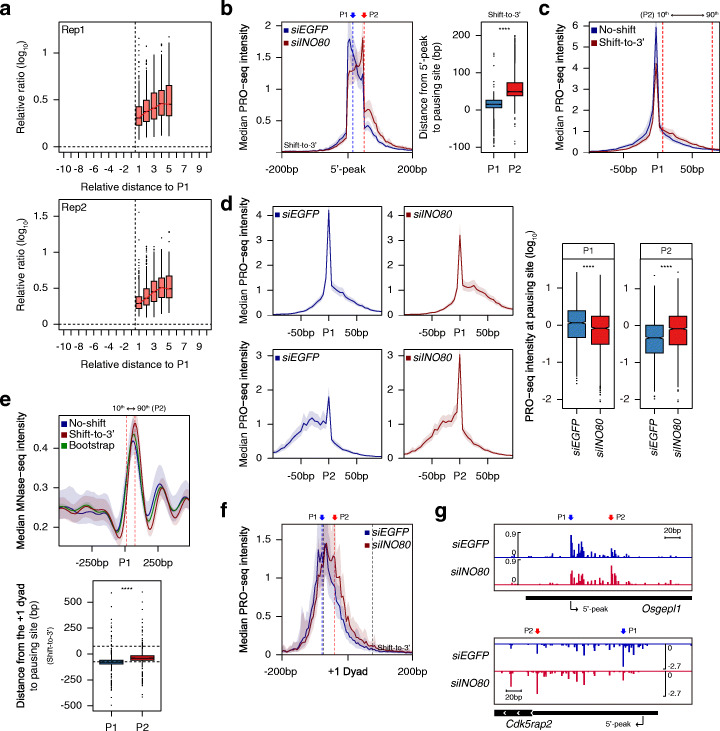
INO80 mediates RNAPII pausing site determination in mESCs. **a** Boxplots represent the relative read ratio (log_10_) at the significantly increasing peaks (the ratio of *siINO80* versus *siEGFP*) in a manner similar to Fig. 3b (2406 peaks for replicate 1 and 2408 peaks for replicate 2 out of 2962 peaks at 1890 genes). **b** Average profile displays median PRO-seq intensity in mESCs treated with *siEGFP* or *siINO80* for shift-to-3′ genes (left). Boxplot shows the distance from 5′-peak to the indicated pausing sites (right). **c** Average profiles exhibit median PRO-seq intensity in *siEGFP*-treated mESCs at no-shift and shift-to-3′ genes. **d** Average profiles depict median PRO-seq intensity (left), and boxplots represent the smoothed PRO-seq intensity (right) at the indicated pausing sites for shift-to-3′ genes. **e** Average profile shows median MNase-seq intensity obtained from untreated mESCs (GSM2906312 and GSM2906313) around P1 of no-shift and shift-to-3′ genes (top). The green line indicates the median of 1000 bootstrap samples (Each sample containing the same number of genes to the original population and was obtained by resampling allowing replacement). Boxplot exhibits the distance from the + 1 dyad to the indicated pausing sites for shift-to-3′ genes (bottom). For (**c**) and (**e**), the two dotted lines represent the 10th (12.9 bp) and 90th (76.1 bp) percentiles of P2 relative to P1. **f** Average profile displays median PRO-seq intensity around the + 1 dyad for shift-to-3′ genes. For (**e**) and (**f**), only genes with nucleosomes overlapped with H3K4me3 ChIP-seq enrichment (GSM590111) were used in an effort to exclude false-positive nucleosomes (431 no-shift genes and 500 shift-to-5′ genes upon INO80-KD). **g** Genome browser view of PRO-seq signal for representative shift-to-3′ genes. All PRO-seq data were generated using combined biological replicates. For average profiles, medians reflect the 5-bp bin (PRO-seq) or the 10-bp bin (MNase-seq). Asterisks represent statistically significant differences based on Mann-Whitney *U* test

Because the PRO-seq signal was highly confined near 5′-peak, we determined regions from 100 bp upstream to 200 bp downstream of 5′-peak as PR regions for mESCs. Based on PRO-seq coverage of these regions, we classified genes as being paused (*N* = 12,988) or not paused (*N* = 2072) among the 16,068 protein-coding genes (considering only the isoform with the highest expression level, if multiple isoforms exist; see “Methods”). To analyze the transition of RNAPII pausing under INO80-KD, we defined P1 and P2 in the same manner as in *S. cerevisiae*. However, results obtained from these analyses differed from those observed in *S. cerevisiae.* We found significantly increasing 2962 peaks for 1890 genes upon INO-KD and examined that increasing peaks were mainly located downstream of P1 (Fig. 5a). We referred to 1658 genes with downstream P2 as “shift-to-3′ genes”, 316 genes with upstream P2 as “shift-to-5′ genes”. For both gene sets, only some of the genes exhibited multiple increasing peaks, so that we chose the increasing peak with the lowest *p* value at those genes as in *S. cerevisiae* (545 genes out of 1658 shift-to-3′ genes and 37 genes out of 316 no-shift genes showed multiple increasing peaks). Heatmaps of the PRO-seq log_2_ fold change around P1 showed that downstream increasing P2 was more robust and frequent than upstream increasing P2 (Additional file 1: Fig. S10c). We additionally designated 1659 genes with non-significantly increasing peaks as “no-shift genes” (Additional file 1: Fig. S10d). We observed a significant increase in PRO-seq signals at P2 for shift-to-3′ genes upon INO80-KD (Fig. 5b), whereas no-shift and shift-to-5′ genes did not exhibit such increase in PRO-seq signal (Additional file 1: Fig. S10e). Thus, we also focused on our analysis for shift-to-3′ genes in mESCs.

We observed that RNAPII at shifted genes uses more alternative pausing sites than not shifted genes as examined in *S. cerevisiae*. Shift-to-3′ genes showed lower PRO-seq enrichment at P1 than no-shift genes and the accumulation of PRO-seq signal downstream of P1 in the *siEGFP*-treated mESCs (Fig. 5c). We further calculated retained ratio at P1 and that of no-shift genes was significantly higher than that of shift-to-3′ genes (Additional file 1: Fig. S10f). In addition, we found that the majority of P2 was located where such accumulation occurs (Fig. 5c; the 10th and 90th percentiles of the relative position of P2 to P1 for shift-to-3′ genes are represented by the dotted lines). This overlapped location indicates that INO80-KD displaces RNAPII to, not random, but particular positions where pausing weakly occurs in the physiological condition. We also examined that the PRO-seq peak at P1 of shift-to-3′ genes was significantly decreased upon INO80-KD. This reduction was accompanied by an increase in the accumulated signal downstream of P1 (Fig. 5d, average profiles, top). Such increase was much more pronounced when aligned at P2 (Fig. 5d, average profiles, bottom). In addition, we observed that the sequence preferences at P1 and P2 of shift-to-3′ genes exhibited a marked similarity and that this preference was uniquely identified at the pausing site as in *S. cerevisiae* (Additional file 1: Fig. S10g), additionally supporting the usage of P2 as the alternative pausing site.

We next analyzed existing MNase-seq data obtained from untreated mESCs [68] to decipher whether these INO80-dependent pausing site determination defects showed any link to nucleosome context in mESCs. We observed better-phased nucleosome architecture around P1 of shift-to-3′ genes than no-shift genes and even bootstrapped estimation (Fig. 5e, top). In addition, at shift-to-3′ genes, + 1 nucleosome dyad tended to be located in much closer proximity to P2 than to P1 (Fig. 5e, bottom). P1 of mammalian shift-to-3′ genes was located near the entrance of + 1 nucleosome; however, the majority of P2 was found between P1 and + 1 dyad. Moreover, we examined the significant transition of PRO-seq distribution toward + 1 nucleosome in shift-to-3′ genes upon INO80-KD (Fig. 5f). Consistently, retained ratio at P1 was significantly decreased upon Ino80-KD, whereas those at P2 showed a significant increase (Additional file 1: Fig. S10h). In control analysis, we detected a non-significant difference in the PRO-seq pattern around + 1 dyad of no-shift genes (Additional file 1: Fig. S10i). Genome browser view of representative shift-to-3′ genes indicated that RNAPII pausing was clearly shifted toward downstream upon INO80-KD (Fig. 5 g).

**Fig. 6:**
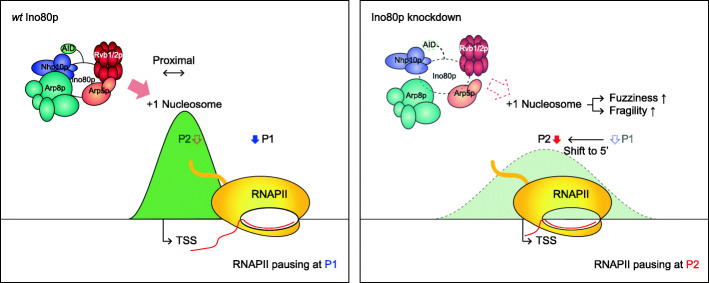
Model of the function of Ino80p in mediating pausing site determination. Model depicts a regulatory role of Ino80p in proper localization of RNAPII pausing in a nucleosome context-dependent manner in *S. cerevisiae*. See “Discussion” for details

Furthermore, we found that occupancy of chromatin-associated INO80, which was analyzed using published ChIP-seq data generated in the J1 cell line mESCs [50], was much higher at shift-to-3′ genes compared to no-shift genes (Additional file 1: Fig. S10j). Consistent with this, de novo motif finding analysis using HOMER [69] indicated that the YY1 motif was significantly enriched in the promoter regions of shift-to-3′ genes relative to no-shift genes (Additional file 1: Fig. S10k). YY1 is physically associated with the mammalian INO80 complex [70], providing additional support for the engagement of INO80 with these genes. We did not observe a similar motif in *S. cerevisiae*, which seems to reflect its lack of an identified homolog for YY1. Specific recruitment of mammalian INO80 to the chromatin through YY1 may result in a stronger association of INO80 with the INO80-dependent genes in mESCs than in *S. cerevisiae*. Based on our collective results, we propose that mammalian INO80 is essential in the proper localization of RNAPII pausing at P1 in a nucleosome context-dependent manner as observed in *S. cerevisiae* (Fig. 6).

## Discussion

Although the previous study reported that promoter-proximal pausing-like distribution was not observed in *S. cerevisiae* [28], however as described above, the investigations from several other studies led to speculation that *S. cerevisiae* may also have a regulatory mechanism governing early transcription elongation. Here, using PRO-seq experiments, we propose that there is widespread promoter-proximal RNAPII enrichments in *S. cerevisiae* whose general features are similar to those in *S. pombe* and metazoans. We revealed highly confined PRO-seq signals immediately downstream of the observed TSS of RNAPII-transcribed protein-coding genes in *S. cerevisiae* (Fig. 2a)*.* However, the promoter-proximal RNAPII elongation in *S. cerevisiae* occurred more broadly, and RNAPII seemed to be paused farther downstream than in metazoans. Indeed, most of the main pausing site (i.e., P1) was located downstream of the + 1 nucleosome (Fig. 2f). This finding was unlike that in higher eukaryotes, where most pausing was found to occur upstream of the + 1 dyad [16, 32, 33]. One of the plausible reasons for this discrepancy could be the difference in promoter structures in *S. cerevisiae* and metazoans. In *S. cerevisiae*, + 1 nucleosome is generally located much more proximal to TSS than in metazoans. The difference in the relative distance between TSS and + 1 nucleosome could physically affect the early elongation of RNAPII. Another one might be that NELF, whose cooperative interaction to the elongation complex with DSIF [71] is involved in RNAPII pausing at a more promoter-proximal region [72], is not conserved in yeasts including *S. cerevisiae* and *S. pombe* [22, 23]. The hypothesis is consistent with the PRO-seq distribution reported in *S. pombe*, which showed a near-overlapping association of RNAPII pausing with a + 1 dyad [28]. Interestingly, across several species, the relative position of + 1 nucleosome to TSS is highly related to the presence of NELF [23].

We examined the marked transition of RNAPII pausing toward + 1 nucleosome upon Ino80p knockdown. This change is closely associated with the regulation of nucleosome distribution around the main pausing site by Ino80p. In *S. cerevisiae*, most of the main pausing site is located downstream of + 1 nucleosome. The loss of Ino80p induced the transition of RNAPII toward upstream, making it closer to + 1 dyad (Fig. 4a, c), accompanied by a moderate increase in nucleosome fuzziness and fragility (Fig. 4b). Consistently, previous studies also reported increased nucleosome fuzziness in Ino80p mutants [67, 73] and examined that Ino80p functions to pull the + 1 nucleosome toward the NFR, leading to the collapsed + 1 nucleosome upon Ino80p knockdown for a subset of genes in *S. cerevisiae* [58]. Altogether, these results suggest that Ino80p depletion enhanced the accessibility of the alternative pausing site (i.e., P2) to RNAPII and that Ino80p plays a role in determining where RNAPII to pause in a nucleosome context-dependent manner (Fig. 6).

We observed that INO80 knockdown also yielded a defect in RNAPII pausing site determination in mESCs. However, the direction of the RNAPII pausing site shift was opposite in the two species. One possible explanation is that Ino80p plays different roles in these species since the Ino80 complex contains species-distinct components that could target Ino80p to specific contexts. Alternatively, our observations of the highly phased nucleosome distribution around the main pausing site and the proximity of the alternative pausing site to + 1 nucleosome in both organisms led us to postulate that the discrepancy in direction could be due to differences in chromatin architectures around promoter regions in *S. cerevisiae* versus mESCs [74, 75]. In mESCs, the main pausing site was located near the entrance of the + 1 nucleosome. We found that the loss of INO80 induced the transition of the pausing site toward downstream that make it closer to the + 1 dyad (Fig. 5e, f) as observed in *S. cerevisiae*. Although we did not analyze the nucleosome distribution upon the loss of INO80 in mESCs, based on the conserveness of its remodeling activity in metazoans [76, 77], we speculate the link between INO80-dependent RNAPII pausing site determination and the regulation of + 1 nucleosome. The collapsed + 1 nucleosome that might be due to INO80 depletion could increase the accessibility of the alternative pausing site, which is around 40 bp upstream of the + 1 dyad (Fig. 5e, bottom; the median of the distance from + 1 dyad to P2), to RNAPII. A previous study implied that the positioned + 1 nucleosome recruits more NELF and enhances promoter-proximal pausing [78]. Thus, mammalian INO80-dependent regulation of + 1 nucleosome may cooperatively work to establish RNAPII pausing through recruiting *trans*-activating pausing factors such as NELF. In support of our hypothesis, a recent study using human NELF-C-AID suggested that NELF loss results in a similar downstream shift of RNAPII pausing accumulated at the 2nd pausing site closely associated with the + 1 nucleosome [33]. However, it is not clear that the increase in PRO-seq signal at the alternative pausing site was caused by the RNAPII populations normally released from the main pausing site. Future studies about the relationship between the INO80-dependent regulation of RNAPII pausing and the premature termination would shed light on the detailed mechanism governing the early elongation in mESCs.

We found genes whose pausing sites are Ino80p-dependently determined in *S. cerevisiae* and mESCs exhibit common features. The nucleosome architecture of those genes showed a highly phased distribution around the main pausing site due to a relatively constant position of the + 1 nucleosome dyad to the main pausing site. In addition, the alternative pausing site is closely located to the + 1 nucleosome. Since there is an increase in RNAPII that overlays with + 1 nucleosome upon the loss of Ino80p in both organisms, one possible explanation is that nucleosome-dependent pausing works in the absence of Ino80p and causes RNAPII pausing at the alternative pausing site. To investigate those Ino80p-dependent genes more precisely, we further examined other features, including the localization of several transcription factors. However, we did not figure out things that distinguish Ino80p-dependent and Ino80p-independent gene groups in both organisms. It suggests that nucleosome context itself is specifically associated with genes bearing alternative pausing sites. Based on these observations, an alternative explanation is that RNAPII pausing at the alternative pausing sites in physiological conditions might be suppressed in a nucleosome context-dependent manner. In line with this, a recent study has suggested that the nucleosomal barrier, especially the + 2 nucleosome, is closely linked to the Spt4p-dependent RNAPII movement in *S. cerevisiae* [79].

## Conclusion

In this study, we report the accumulated early elongating RNAPII within promoter-proximal regions in *S. cerevisiae* using PRO-seq experiments. These RNAPII enrichments exhibit both similar and different attributes to that of promoter-proximal RNAPII pausing in *S. pombe* and metazoans. Furthermore, we find that RNAPII pauses at alternative pausing sites for a subset of genes in both *S. cerevisiae* and mESCs. Genes with alternative pausing sites exhibit a highly phased nucleosome distribution around main pausing sites, and alternative pausing sites are closely associated with + 1 nucleosome. Ino80p depletion causes the accumulation of RNAPII at alternative pausing sites accompanied by the disruption of the + 1 nucleosome. Based on the collective results, we hypothesize that the highly conserved Ino80 chromatin remodeling complex mediates RNAPII pausing site determination. Our works further demonstrate the link between early transcription elongation and nucleosome and provide evidence that chromatin remodelers could play a role in regulating promoter-proximal RNAPII pausing.

## Methods

### Yeast strains and cell culture

The budding yeast strains used in this study are listed in Table S2 (Additional file 1). AID-tagged proteins were conditionally depleted using 250 mM auxin (Sigma, I2886) stock dissolved in ethanol at a final concentration of 0.5 mM, as previously described [52]. Briefly, Ino80p-AID cells were grown to the mid-log phase at 30 °C in YPD containing ethanol. The ethanol was removed by media exchange, and cells were incubated with auxin (where indicated) for 3 h to allow conditional depletion. The auxin was removed by media exchange, and cells were incubated in an auxin-free medium for an additional 3 h. At the indicated time points, Ino80p-AID cells were harvested and subjected to PRO-seq and PRO-cap. The workflow is schematically presented in Fig. S7a (Additional file 1). The efficiency of Ino80p knockdown was confirmed by Western blotting. To generate the deletion strains, we conducted standard LiAc transformation using PCR-based gene targeting. These cells were incubated to the mid-log phase at 30 °C in YPD and were subjected to PRO-seq. For fission yeast, cells were incubated to the mid-log phase at 30 °C in yeast extract with supplements (YES).

### mESC culture

E14Tg2a mESCs were maintained under feeder-free conditions at 37 °C with 5% CO_2_ in humidified air. Briefly, mESCs were cultured on gelatin-coated dishes in Glasgow’s minimum essential medium (GMEM) containing 10% knockout serum replacement (Gibco, 10828-028), 1% non-essential amino acids (Gibco, 11140-050), 1% sodium pyruvate (Gibco, 11360-070), 0.1 mM β-mercaptoethanol (Gibco, 21985-023), 1% FBS (Hyclone, SH30917.03), 0.5% antibiotic-antimycotic (Thermo, 15140122), and 1000 units/ml LIF (Millipore, ESG1106).

### RNA interference

The siRNAs against EGFP (5′-GUUCAGCGUGUCCGGCGAG-3′) and INO80 (5′-GGCUUAUCUGUAAAGGCACAAUUGA-3′) were synthesized and annealed by Bioneer. mESCs were seeded to plates, incubated for 24 h, and transfected with the indicated siRNAs (final concentration, 50 nM) using DharmaFECT I (Dharmacon, T-2001-03) according to the manufacturer’s protocol. Briefly, 50 nM of siRNAs and DharmaFECT I diluted in Opti-MEM (Gibco, 31985062) were separately incubated for 5 min at 25 °C, and further mixed and incubated for 20 min at 25 °C, and then used for transfection. The culture medium was replaced at 24 h of transfection, and cells were harvested at 48 h of transfection.

### Western blot analysis of protein depletion

Whole-cell lysates of Ino80p-AID cells were prepared using a standard bead-beating protocol, and proteins were eluted by boiling at 100 °C for 5 min in 2× SDS sample buffer (20% glycerol, 0.4% bromophenol blue, 100 mM Tris-Cl, pH 6.8, 4% SDS, and 200 mM β-mercaptoethanol). The utilized antibodies included anti-FLAG M2 (Sigma A8592; used at 1:3000) and anti-β-actin (Santa Cruz sc-47778 HRP; used at 1:5000). Anti-FLAG M2 was used to detect 9xFLAG-tagged Ino80p, and β-Actin was used as a loading control. The Ino80p-AID cells were a gift from the Friedman lab as described above [52].

mESCs were washed with PBS (Welgene, LB004-02) and detached from the dishes by incubation with trypsin-EDTA (Gibco, 25300-054) at 37 °C for 2 min. The trypsin was inactivated by the addition of GMEM with 1% FBS, and 0.5% antibiotic-antimycotic, and the cells were harvested, washed with PBS, and resuspended in EBC300 (120 mM NaCl, 0.5% NP-40, and 50 mM Tris-Cl, pH 8.0) containing protease inhibitors. Whole-cell lysates were prepared by vigorous vortexing the cell mixture for 30 min followed by centrifugation for 30 min at 4 °C. Proteins were eluted by boiling at 100 °C for 5 min with 5× SDS sample buffer. The utilized antibodies included anti-INO80 (Abcam, ab118787; used at 1:1000) and anti-Tubulin (Cell Signaling, 2144S; used at 1:4000). Tubulin was used as a loading control.

### Yeast cell permeabilization

Yeast cells were permeabilized as described [17] with some previously reported modifications [80]. Briefly, exponentially growing yeast cells were harvested by centrifugation at 2000 rpm for 3 min at 4 °C. Cells were washed once with ice-cold DEPC-H_2_O. Cell pellets were resuspended in 10 ml of 0.5% sarkosyl (Sigma, L5777) and incubated for 20 min on ice. Cells were spun down at 400×*g* for 5 min at 4 °C and resuspended in storage buffer (10 mM Tris-Cl, pH 8.0, 25% glycerol, 5 mM MgCl_2_, 0.1 mM EDTA, and 5 mM DTT) to an optical density (OD) of 5 per 200 μl. The solutions were flash-frozen using LN_2_ and stored at − 80 °C.

### Isolation of nuclei

mESCs were transfected with the indicated siRNAs, and nuclei were isolated as previously described [15, 81] with some modifications. Briefly, ~ 20 × 10^6^ plated mESCs were washed once with PBS and detached by incubation with trypsin-EDTA at 37 °C for 2 min. The trypsin was inactivated by the addition of GMEM with 1% FBS and 0.5% antibiotic-antimycotic, and the cells were harvested and washed twice with ice-cold PBS. The cells were resuspended in 5 ml of ice-cold swelling buffer (20 mM Tris-Cl, pH 7.5, 2 mM MgCl_2_, 3 mM CaCl_2_, and 2 U/ml RNase inhibitor) for 5 min on ice. Lysis buffer (5 ml; 20 mM Tris-Cl, pH 7.5, 2 mM MgCl_2_, 3 mM CaCl_2_, 0.5% NP-40, 10% glycerol, and 2 U/ml RNase inhibitor) was added, and the cell pellets were resuspended by gentle pipetting using an end-cut tip. The cells were centrifuged at 1000×*g* for 5 min at 4 °C, and the cell pellets were resuspended in 1 ml of freezing buffer (50 mM Tris-Cl, pH 8.3, 40% glycerol, 5 mM MgCl_2_, and 0.1 mM EDTA). The pelleted nuclei were transferred into a new 1.5-ml tube and were resuspended in a freezing buffer at ~ 5 × 10^6^ nuclei per 100 μl. The solutions were flash-frozen using LN_2_ and stored at − 80 °C.

### PRO-seq and PRO-cap library preparation

Nuclear run-on reactions and RNA extractions were performed based on the published protocol [17] with minor modifications previously reported [80, 81]. Briefly, the flash-frozen yeast cells were quickly thawed on ice. For the yeast spike-in control, 0.125 OD of permeabilized *S. pombe* (ED665) cells was added to each 5 OD of permeabilized *S. cerevisiae* sample (or vice versa) before the nuclear run-on reaction was performed. Combined yeast cells were spun down at 400×*g* for 5 min at 4 °C. Nuclear run-on reactions were conducted with 25 μM biotin-11-UTP (PerkinElmer, NEL543001EA), 25 μM biotin-11-CTP (PerkinElmer, NEL542001EA), 125 μM ATP (Roche, 11140965001), and 125 μM GTP (Roche, 11140957001) in run-on reaction buffer (20 mM Tris-Cl, pH 7.7, 200 mM KCl, 5 mM MgCl_2_, 2 mM DTT, and 0.4 U/μl RNase inhibitor) with 0.5% sarkosyl. The reaction mixtures were incubated at 30 °C for 5 min. For the isolated nuclei of mESCs, nuclear run-on reactions were performed with 25 μM biotin-11-UTP (PerkinElmer, NEL543001EA), 25 μM biotin-11-CTP (PerkinElmer, NEL542001EA), 125 μM ATP (Roche, 11140965001), and 125 μM GTP (Roche, 11140957001) in run-on reaction buffer (5 mM Tris-Cl, pH 8.0, 150 mM KCl, 2.5 mM MgCl_2_, 0.5 mM DTT, and 0.4 U/μl RNase inhibitor) with 0.5% sarkosyl. The reaction mixtures were incubated at 37 °C for 5 min.

RNA was extracted from the run-on-reacted cell pellets using either a standard hot-phenol method (for yeast samples) or TRIzol LS (Ambion, 10296028; for mESC samples). Next, the respective library was generated, followed by the published PRO-seq or PRO-cap protocols [17] for the steps spanning RNA fragmentation by base hydrolysis to full-scale PCR amplification. Note that there were a few differences in the applied reagents: We used Superscript IV reverse transcriptase (Invitrogen, 18091050) instead of Superscript III (Invitrogen, 56575); we used 25 mM of each dNTP (Thermo Scientific, R1121) instead of 12.5 mM of each dNTP (Roche, 03622614001); and we used Phusion High-Fidelity DNA Polymerase (Thermo Scientific, F530L) instead of Phusion polymerase (NEB, M0530). DNA libraries of ~ 100 bp to 350 bp were selected by agarose gel extraction (Zymo Research, D4007) according to the manufacturer’s protocol and sequenced using an Illumina HiSeq X Ten, HiSeq 4000, and NovaSeq 6000.

### Sequence alignment and data processing (PRO-seq and PRO-cap)

Sequence alignment and data processing were performed based on the publicly available alignment pipelines of GitHub, as used in the previous study [80] with minor modifications. Briefly, raw sequencing reads were processed using FASTX-Toolkit (http://hannonlab.cshl.edu/fastx_toolkit/) as follows: Adaptor sequences (5′-TGGAATTCTCGGGTGCCAAGG-3′) were removed, the reads were trimmed to a maximum length of 36 bp and, for PRO-seq, the reads were reverse-complemented. Next, reads that mapped to rRNA sequences were depleted using SortMeRNA [82], and reads that were not mapped to rRNA sequences were uniquely aligned to the genome using Bowtie, with the algorithm allowing for two mismatches [83]: The processed reads of yeast samples that were generated with the spike-in approach were mapped to a combined genome consisting of *S. cerevisiae* (sacCer3) and *S. pombe* (SpombeASMv2), and then uniquely aligned reads from each genome were parsed for downstream analysis. The processed reads of mESCs samples were mapped to the *M. musculus* mm10 genome. The coverage of the aligned reads was generated using the genomecov function of BEDtools [84]. Only the most 3′ nucleotide of each read was calculated for PRO-seq, and only the most 5′ nucleotide of each read was calculated for PRO-cap. For the spike-in control, the recorded coverage in the bedGraph file was normalized to the relative number of uniquely mapped spike-in counts, and these normalized reads were counted in reads per million mapped reads (RPM) considering the sequencing depth of experimental reads. For mESCs data, which was generated without the spike-in control, the reads were presented in RPM. The bedGraph file was ultimately converted to the BigWig file by bedGraphToBigWig [85]. The downstream analysis was performed based on the publicly available custom R scripts on GitHub, as previously reported [80].

Protein-coding genes based on the annotated data in the Saccharomyces Genome Database (SGD; *N* = 6692) were initially used for *S. cerevisiae* samples*.* The observed TSS was defined as the single nucleotide with the most PRO-cap reads within the 250 bp upstream and downstream of the annotated TSS, in a similar manner to that used in the previous report [28]. Genes with no PRO-cap signal, genes that had PRO-seq reads lower than 10, genes shorter than 300 bp, and 18 mitochondria-encoded genes were filtered out; in the end, 5697 genes were used out of 6692 SGD genes. For mESCs, protein-coding genes based on the RefSeq annotation were downloaded from UCSC Genome Browser. For genes with multiple isoforms, only the isoform with the highest expression level was considered. Genes with the PRO-seq reads lower than 10 and those shorter than 1 kb were discarded; in the end, 16,068 genes were selected out of 38,984 genes. The annotated TSS ± 1 kb was tiled in a 50-bp window by shifting 5 bp, and the PRO-seq coverage for each window was measured. The window of the most PRO-seq reads was selected, and the 5′ position of the selected window was referred to as the “5′-peak,” in a manner similar to that described in a previous study [15]. To identify paused gene sets, the pausing index (PI) was first calculated as a ratio of promoter-proximal (PR) density to gene body (GB) density. PRO-seq density was calculated as the number of sense strand PRO-seq reads per mappable bases within the indicated region as described in the previous study [15, 28]. Mappable bases indicated the bases that are uniquely aligned when aligning all possible 36-mer sequences of the genome to the same genome. For *S. cerevisiae*, the regions from the observed TSS to downstream 250 bp (TSS to TSS + 250 bp) were used as the PR regions, and those from TSS + 250 bp to the annotated TES were used to calculate the GB signals. For mESCs, the regions from upstream 100 bp to downstream 200 bp of the 5′-peak were used as the PR regions, and the regions downstream 1 kb from the 5′ peak to the annotated TES were used as the GB regions. To classify the genes as being paused or not paused, we calculated the significance of PI. Significant enrichments of the signals from PR regions compared to GB regions were evaluated using Fisher’s exact test with Bonferroni’s correction as in the previous study [28]. Briefly, a gene was identified as being paused if the *p* value was lower than 0.01 and as being not paused if the *p* value was higher than 0.99, as determined using combined replicates. A gene exhibiting *p* value < 0.05 or *p* value > 0.95 for both biological replicates was further assigned as a high-confidence paused or high-confidence not paused gene, respectively. All average profiles centered on the indicated point in this work were generated using a bootstrapped estimation. Briefly, 1000 random gene sets were taken as each representing 10% of the total genes, and the median and confidence intervals were calculated using each average of the subsample. In the relevant figures, the thick line represents the median value and shaded regions indicate the 12.5th and 87.5th percentiles.

### Identification of increasing peaks upon knockdown or deletion

We first smoothed each PRO-seq data with a Savitzky-Golay smoothing method [60] using the R sgolayfilt function (*p* = 4 and *n* = 7). For each gene, we then identified consensus peaks present across all samples in the data set and filtered out non-systematic peaks with 3-folds lower than the mean value of promoter-proximal signals. The peak showing the highest mean smoothed read count for two replicates in the control condition (e.g., Ctrl or *wt*) was designated as P1. We calculated the smoothed read count ratios of the other peaks to P1, resulting in a *n* x *m* read count ratio matrix for *m* samples and *n* consensus peaks identified from all genes. To identify the increasing peaks, we next applied an empirical statistical test previously reported [61] to the *n* × *m* read count matrix. Briefly, for each gene, we calculated *t*-statistic values for the observed ratios of consensus peaks in the samples between two conditions (e.g., Ctrl vs. KD). We then generated an empirical null distribution for *t*-statistic values by calculating *t*-statistic values after randomly permuting the samples in the *n* × *m* read count matrix 1000 times. The adjusted *p* value was calculated by performing the left-sided test for its observed read count ratio using the empirical distribution. Finally, we selected the increasing peaks as the ones with a *p* value < 0.1. We further filtered out false-positive peaks with log_2_ median ratios lower than a cutoff after estimating an empirical null distribution of log_2_ median ratios for the smoothed read count ratios between the two conditions during the above random permutations. The cutoff was determined as the mean of 10th and 90th percentiles in the empirical distribution. To select genes with the unchanged peaks upon knockdown or deletion, we first excluded genes containing the selected increasing peaks as described above. We then sorted the remaining peaks in descending order of *p* value and selected *n* peaks from the top to set the number of genes similar to those of the genes with increasing peaks to be compared.

### Analysis of publicly distributed ChIP-seq and MNase-seq data

Raw sequencing reads of the indicated accession numbers were downloaded from NCBI GEO unless otherwise noted. For MNase-seq, raw reads were uniquely mapped to the *S. cerevisiae* sacCer3 genome or to the *M. musculus* mm10 genome using Bowtie, which trimmed the 3′ bases to 36 bp (if the raw reads were longer than 36 bp), allowed two mismatches, and for paired-end data, restricted the maximum insert size to 200 bp. BEDtools was used to covert the aligned BAM files to BED formats [84]. The BED files were then processed by iNPS [57] to determine the nucleosome positions. Briefly, the “MainPeak” nucleosome that was the closest to either the observed TSS in *S. cerevisiae* or the observed 5'-peak in mESCs was assigned as the + 1 nucleosome. The + 1 dyad was defined as the mid-point between the start and the end inflection, and 75 bp around the + 1 dyad was referred to as the + 1 nucleosome position. To discard false-positive nucleosome positions, nucleosomes that did not overlap the H3K4me3 ChIP-seq enrichment calculated from the existing data [64, 86] were discarded, as previously reported [33]. The Gaussian smoothing value was used to process BigWig files, and reads were normalized to the total mapped reads (single-end data) or the total number of valid pairs (paired-end data). For ChIP-seq, a combined genome consisting of *S. cerevisiae* (sacCer3) and *S. pombe* (SpombeASMv2) was used, and unique reads from each genome were parsed for downstream analysis. MACS2 [87] was used to convert the aligned BAM files to bedGraph formats. For the spike-in control, the recorded coverage in the bedGraph file was normalized to the relative number of uniquely mapped spike-in counts, and these normalized reads were counted in RPM considering the sequencing depth of experimental reads. The bedGraph file was ultimately converted to the BigWig file by bedGraphToBigWig [85].

## Supplementary Information

**Additional file 1 **: **Fig. S1**. PRO-seq analysis in *S. cerevisiae* upon the loss of Spt4p. **Fig. S2**. PRO-seq analysis in *S. pombe.* **Fig. S3**. PRO-seq analysis in mESCs. **Fig. S4**. PRO-cap detects the precise transcription initiation sites genome-wide. **Fig. S5**. PRO-seq is highly correlated with Rpb3p NET-seq and ChIP-exo in *S. cerevisiae.* **Fig. S6**. Correlation of promoter-proximal PRO-seq pattern with nucleosome architecture and gene activity. **Fig. S7**. AID system is employed to investigate the immediate effect upon Ino80p knockdown. **Fig. S8**. The transition of RNAPII in Ino80p knockdown is independent of both TSS usage and H2A.Z^Htz1^. **Fig. S9**. The Ino80 complex is essential for RNAPII pausing site determination associated with the + 1 nucleosome. **Fig. S10**. INO80 knockdown yields RNAPII pausing site determination defect in mESCs. **Table S1**. Summary of PRO-seq reads and reproducibility obtained in this study. **Table S2**. List of *S. cerevisiae* strains used in this study.

**Additional file 2.** Review history.

## Data Availability

The raw and processed PRO-seq and PRO-cap data produced in this paper have been deposited at Gene Expression Omnibus (GEO) under the accession number GSE158622 [88]. All other publicly available genome-wide data have been retrieved from GEO under the accession number GSE76142 [89] (PRO-seq in *S. cerevisiae* and *S. pombe*), GSE25107 (Rpb3p NET-seq in *S. cerevisiae*) [90], GSE87657 (Rpb3p ChIP-exo in *S. cerevisiae*) [91], GSE58859 (BioGro in *S. cerevisiae*) [92], GSE118214 [93] (MNase-seq in *S. cerevisiae*) and GSE115412 [94] (MNase-seq and TBP and pSer5 ChIP-seq and Ino80p ChEC-seq in *S. cerevisiae*), GSE95356 [95] (H3K4me3 ChIP-seq in *S. cerevisiae*), GSE130691 [96] (PRO-seq in mESCs), GSE96688 [97] (MNase-seq in mESCs), GSE23943 [98] (H3K4me3 ChIP-seq in mESCs), and GSE49137 [99] (INO80 ChIP-seq in mESCs).
