# Supplementary material for: The chromatin remodeler Ino80 mediates RNAPII pausing site determination
Source: Genome Biol. 2021 Oct 18;22:294. doi: 10.1186/s13059-021-02500-1 (PMC8524862; doi:10.1186/s13059-021-02500-1)
Supplement: Supplementary file 1 — Additional file 1 : Fig. S1. PRO-seq analysis in S. cerevisiae upon the loss of Spt4p. Fig. S2. PRO-seq analysis in S. pombe. Fig. S3. PRO-seq analysis in mESCs. Fig. S4. PRO-cap detects the precise transcription initiation sites genome-wide. Fig. S5. PRO-seq is highly correlated with Rpb3p NET-seq and ChIP-exo in S. cerevisiae. Fig. S6. Correlation of promoter-proximal PRO-seq pattern with nucleosome architecture and gene activity. Fig. S7. AID system is employed to investigate the immediate effect upon Ino80p knockdown. Fig. S8. The transition of RNAPII in Ino80p knockdown is independent of both TSS usage and H2A.ZHtz1. Fig. S9. The Ino80 complex is essential for RNAPII pausing site determination associated with the + 1 nucleosome. Fig. S10. INO80 knockdown yields RNAPII pausing site determination defect in mESCs. Table S1. Summary of PRO-seq reads and reproducibility obtained in this study. Table S2. List of S. cerevisiae strains used in this study. [file 13059_2021_2500_MOESM1_ESM.docx]

**Additional File 1**


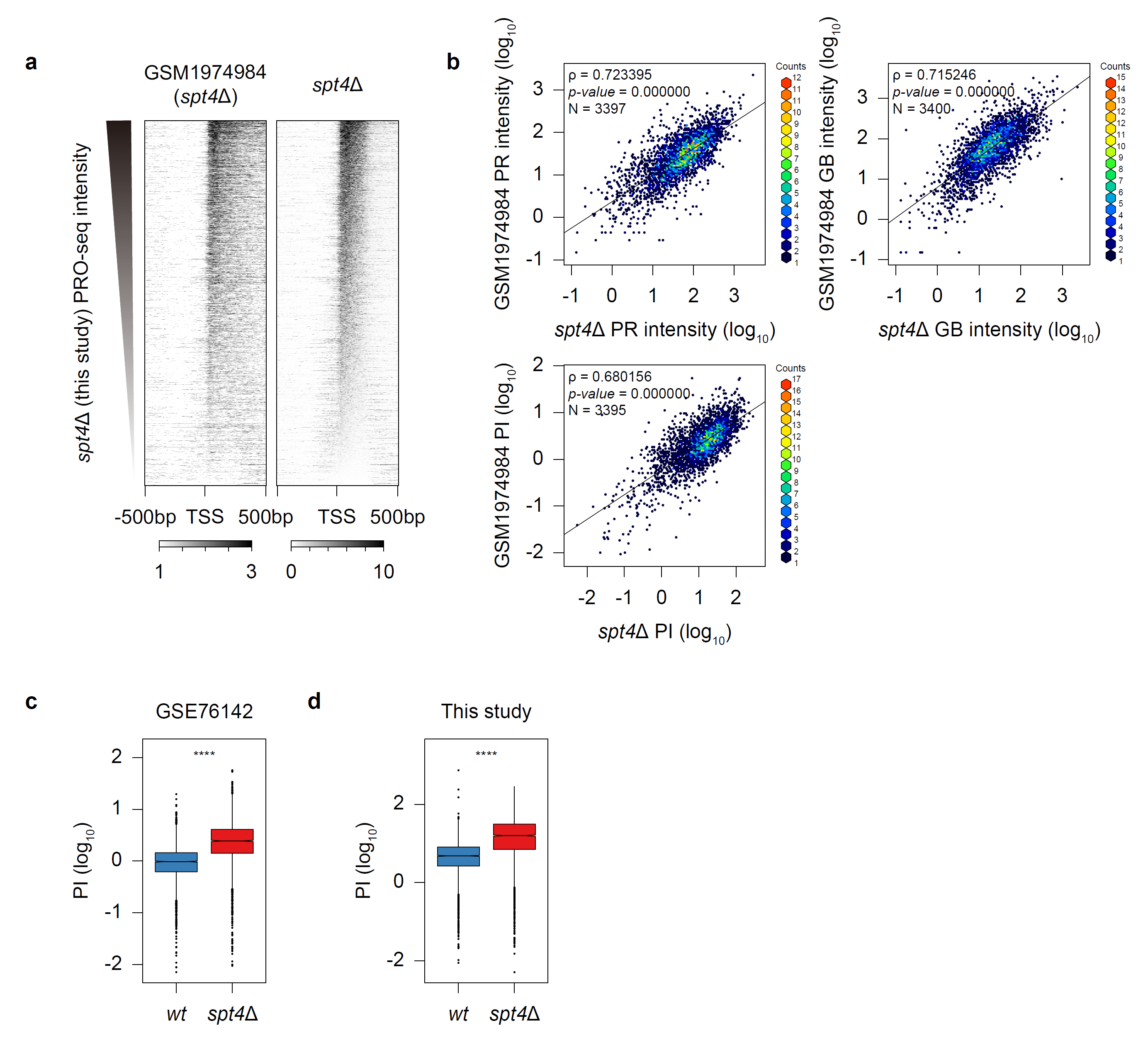


**Fig. S1. PRO-seq analysis in *S. cerevisiae* upon the loss of Spt4p.**

**a** Heatmaps display our PRO-seq data and the previously published PRO-seq data (GSM1974984) generated in *spt4*Δ cells. Genes were sorted by the PR intensity of our *spt4*Δ PRO-seq data. Signals reflect the 10-bp bin. **b** Scatterplots represent a correlation of PR and GB intensity and PI between the indicated data. **c**, **d** Boxplots indicate PI of the indicated samples generated in the previous study (**c**; *wt* is GSM1974983 and *spt4*Δ is GSM1974984) or this study (**d**).

PRO-seq data were generated using combined biological replicates.

**Fig. S2. PRO-seq analysis in *S. pombe*.**

The gene set used in the previous study [1] was used (N = 3,214) for analysis. **a** Heatmaps show our *wt* (ED665) PRO-seq data and the previously published *wt* (972) PRO-seq data (GSM1974985) around TSS. Genes were sorted by the PR intensity of our ED665 PRO-seq data. Signals reflect the 10-bp bin. **b** Scatterplots represent the correlation of PR and GB intensity or PI between the indicated data. **c**, **d** Average profiles represent the median intensity of GSM1974985 (**c**) or ED665 (**d**) data. Genes were grouped into quartiles based on PI of ED665 (**c**) or GSM1974985 (**d**) data, where Q1 represents the highest PI. **e** Venn diagram indicates the overlap between paused and not paused genes defined based on GSM1974985 and ED665 data. *P-value* was calculated using the hypergeometric distribution. **f**, **g** Average profiles display the paused and not paused genes defined based on GSM1974985 (left; 680 paused genes and 2074 not paused genes) or ED665 (right; 1580 paused genes and 1161 not paused genes) data. The green line in the GSM1974985 plot (**f**) indicates 993 genes defined as paused genes in only ED665 data. The green line in the ED665 plot (**g**) represents 587 genes defined as paused genes in both data.

PRO-seq data were generated using combined biological replicates. For average profiles, medians reflect the 10-bp bin.

**Fig. S3. PRO-seq analysis in mESCs.**

All protein-coding genes based on the RefSeq were used (N = 38,943). **a** Heatmaps display our PRO-seq data generated in *siEGFP*-treated E14Tg2a mESCs and the previously published PRO-seq data generated in the 129 cell line of mESCs (GSM3747824, GSM3747825, and GSM3747826). Genes were sorted by the PR intensity of our *siEGFP*-treated PRO-seq data. Signals reflect the 10-bp bin. **b** Scatterplots represent the correlation of PR and GB intensity and PI between the indicated data. **c**, **d** Average profiles represent the median intensity of the previous data (**c**) or our *siEGFP*-treated data (**d**). Genes were grouped into quartiles based on PI of our data (**c**) or the previous data (**d**), where Q1 represents the highest PI. Genes with no mappable reads within PR or GB regions were excluded. The previous data were generated using the combined biological triplicates, and our PRO-seq data were generated using combined biological replicates. For average profiles, medians reflect the 10-bp bin.

**Fig. S4. PRO-cap detects the precise transcription initiation sites genome-wide.**

**a** Average profiles of median PRO-seq (blue) or PRO-cap (red) intensity centered on the identified observed TSS of filtered protein-coding genes (N = 5,697). Medians reflect the 10-bp bin. **b** Sequence logos at the observed TSS were generated using WebLogo [2]. Due to the limited number of inputs, 5,000 genes were randomly selected out of 5,697 genes.

All data were generated using combined biological replicates.


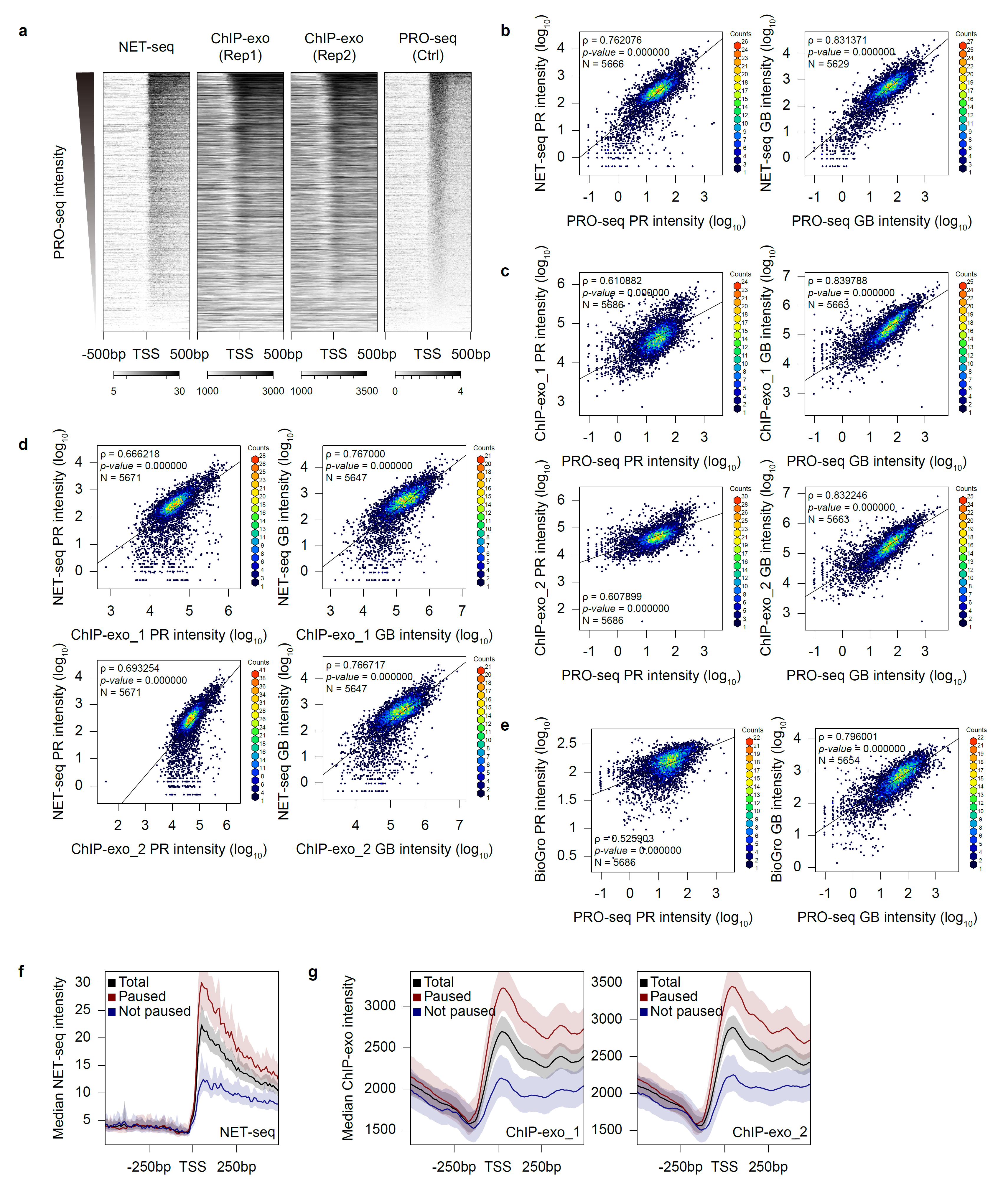
**Fig. S5. PRO-seq is highly correlated with Rpb3p NET-seq and ChIP-exo in *S. cerevisiae.***

**a** Heatmaps show Rpb3p NET-seq (GSM617027), ChIP-exo (Rep1, GSM2337894 and Rep2, GSM2337895), and our Ctrl PRO-seq data at the total filtered protein-coding genes (N = 5,697). Genes were sorted by the PR intensity of our Ctrl PRO-seq data. **b-e** Scatterplots represent the correlation of PR and GB intensity between the indicated data (For BioGro data, GSM1420968 was used). **f, g** Average profiles display median intensity of NET-seq (**f**) or ChIP-exo (**g**) at TSS of total (N = 5,697), paused (N = 2,599), and not paused (N = 1,990) genes.

For the existing data set, processed files downloaded from the NCBI Gene Expression Omnibus (GEO) were used for this analysis. For heatmaps and average profiles, signals and medians reflect the 10-bp bins.

**Fig. S6. Correlation of promoter-proximal PRO-seq pattern with nucleosome architecture and gene activity.**

**a** Heatmaps display our PRO-seq, PRO-cap, existing MNase-seq (GSM3304635), TBP ChIP-seq (GSM3452564), and pSer5 ChIP-seq (GSM3452562) signals around TSS of paused (top) and not paused genes (bottom). Genes were sorted by the PR intensity of our PRO-seq data. PRO-cap intensity reflects regions 250 bp around TSS (TSS -125 bp to TSS +125 bp). **b** Boxplots exhibit the relationship of PRO-seq PR density (left) and PI (right) to the bottom (0-20%), middle (40-60%), and top (80-100%) pentiles of gene activity. All filtered protein-coding genes (N = 5,697) were considered.

Our PRO-seq and PRO-cap samples were generated in the Ctrl condition and the data was generated using combined biological replicates.

**Fig. S7. AID system is employed to investigate the immediate effect upon Ino80p knockdown.**

**a** Schematic illustration of experimental outline. Ino80p-AID cells [3] were grown to the mid-log phase in YPD containing ethanol (Ctrl). The ethanol was washed away, and the cells were incubated with auxin (0.5 mM) for 3 hr (KD). The auxin was washed away, and the cells were incubated without auxin for an additional 3 hr (Rescue). PRO-seq was performed at each indicated time point. **b** Western blot of whole-cell lysates from Ino80p-AID cells under the Ctrl, KD, and Rescue conditions shows that Ino80p was almost completely degraded after 3 hr of auxin incubation and restored after 3 hr of auxin withdrawal. β-Actin was used as a loading control.


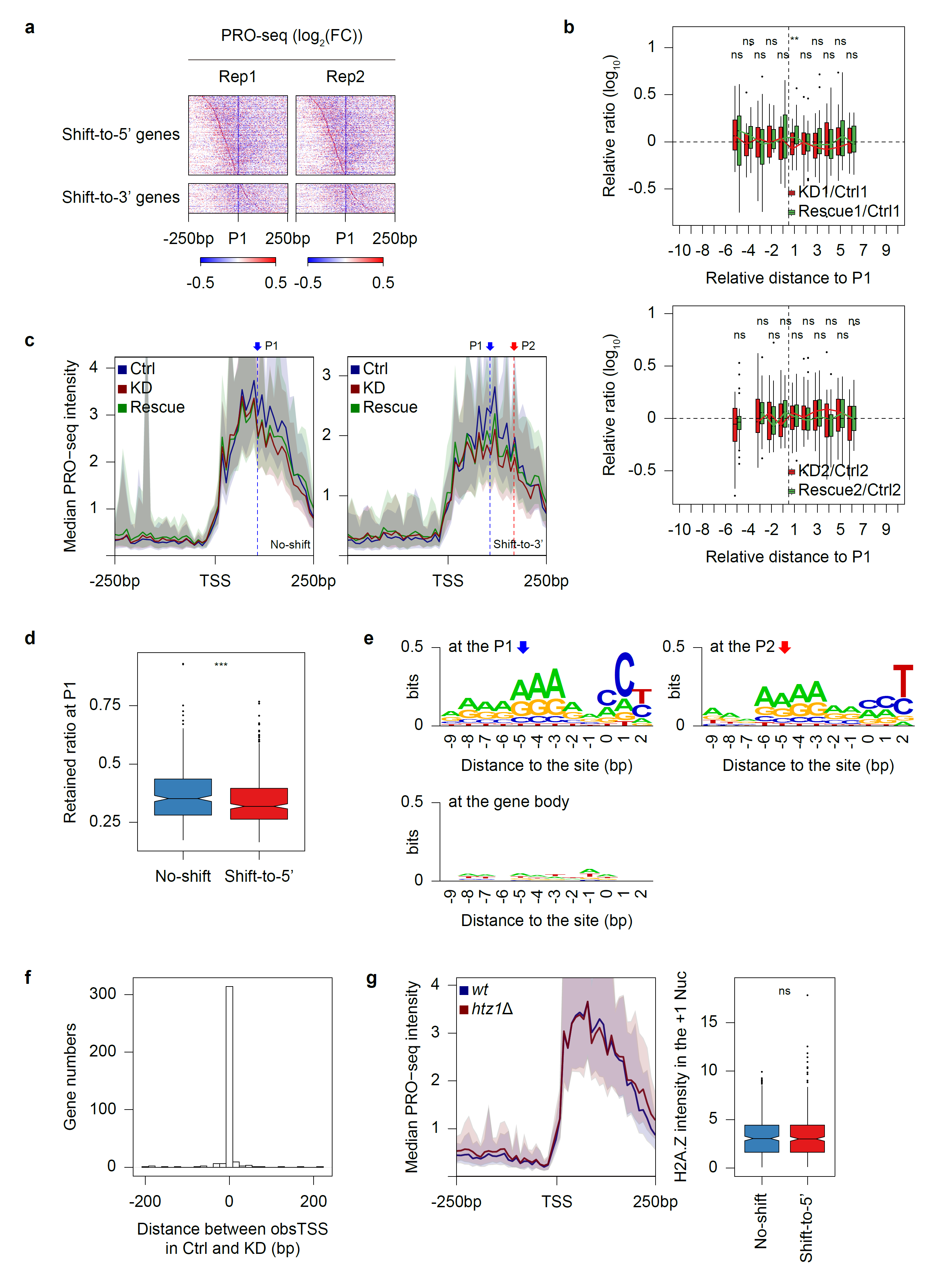


**Fig. S8. The transition of RNAPⅡ in Ino80p knockdown is independent of both TSS usage and H2A.Z^Htz1^.**

**a** Heatmaps display PRO-seq signal upon Ino80p-KD as a log_2_ fold change relative to the Ctrl samples at P1 for each replicate. Genes were sorted by the distance from P1 to P2. **b** Boxplots represent the relative read ratio (log_10_) at the non-significantly increasing peaks (red indicates the ratio of KD versus Ctrl and blue indicates the ratio of Rescue versus Ctrl) in a manner similar to Fig. 3b (285 peaks for replicate 1 and 271 peaks for replicate 2 out of 380 peaks at 350 genes). **c** Average profiles show median PRO-seq intensity around TSS of no-shift and shift-to-3’ genes. The dotted lines represent the median of P1 (blue) or P2 (red) sites. **d** Boxplot exhibits the ratio of smoothed PRO-seq intensity at the peaks corresponding to P1 to those sums of all peaks within PR regions. **e** Sequence logos at P1, P2, and the middle of the GB (gene body) regions for shift-to-5’ genes were generated using WebLogo [2]. **f** Histogram analyzes the distance between the observed TSS for shift-to-5’ genes under Ctrl and KD conditions. A large fraction of genes (85.8%, 303 out of 353) showed no change in their major initiation sites upon Ino80p-KD. **g** Average profile indicates median PRO-seq intensity in *wt* and *htz1*Δ cells for shift-to-5’ genes (left). Boxplot represents the H2A.Z^Htz1^ intensity (GSM2790633, GSM2790634, GSM2790635, and GSM2790636; IP versus input in RPM) at the +1 nucleosome of no-shift and shift-to-5’ genes (right).

PRO-seq data in **a**, **b** was generated using individual biological replicates, and PRO-seq data in **c**, **d**, and **g** was generated using combined biological replicates. For heatmaps, signals reflect the 1-bp bin. For average profiles, medians reflect 10-bp bins. Asterisks represent statistically significant differences, as calculated using either Wilcoxon Signed Rank Test or Mann-Whitney U Test.


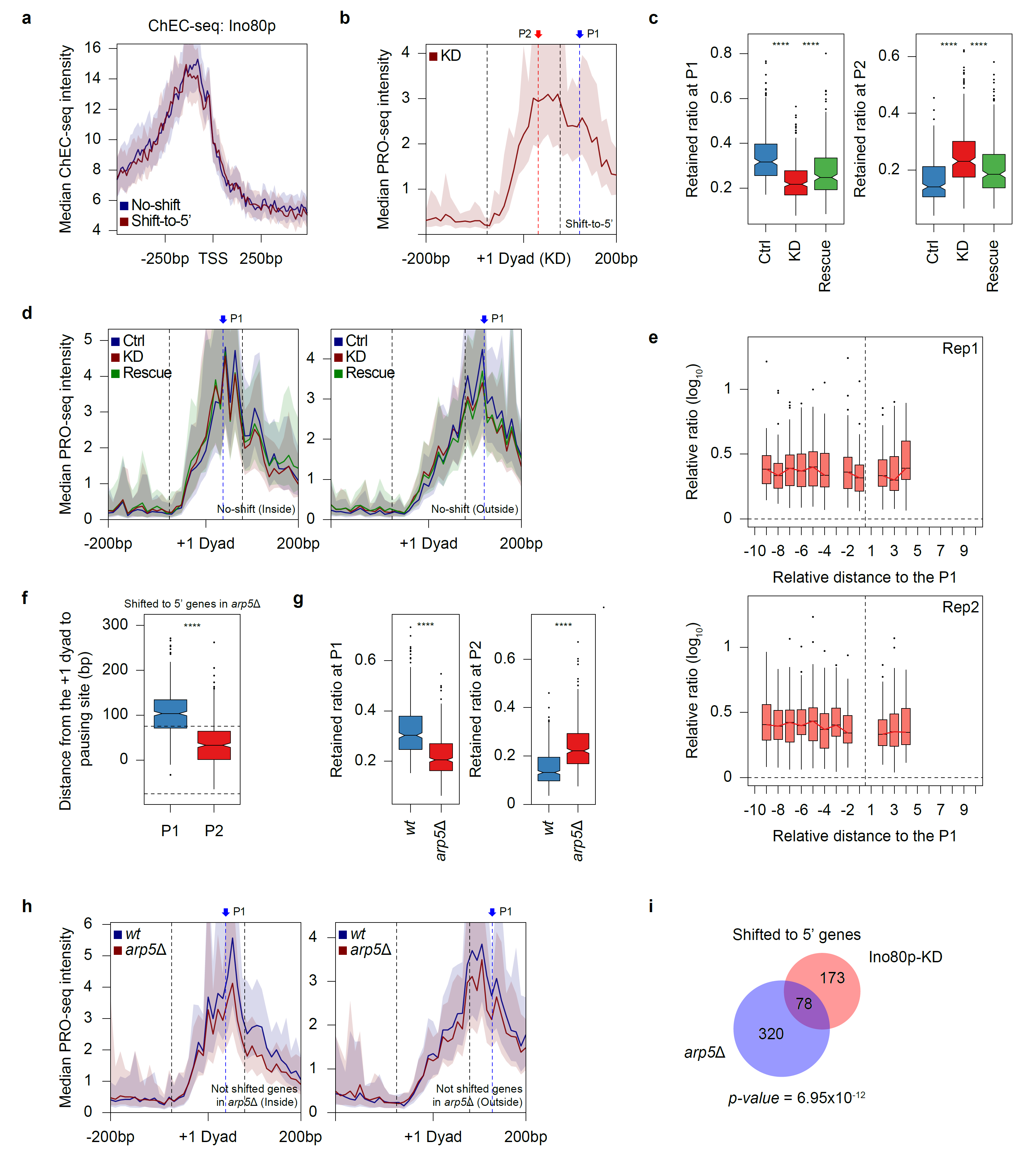


**Fig. S9. The Ino80 complex is essential for RNAPⅡ pausing site determination associated with the +1 nucleosome.**

**a** Average profile displays median ChEC-seq intensity in untreated condition (GSM3452519) at TSS of no-shift and shift-to-5’ genes. A processed sequencing file downloaded from the NCBI Gene Expression Omnibus was used. **b** Average profile shows median PRO-seq intensity of KD samples around the +1 dyad that was defined using MNase-seq under Ino80p-KD (GSM3304637). **c**, **g** Boxplots exhibit the ratio of smoothed PRO-seq intensity at the peaks corresponding to the indicated pausing sites to those sums of all peaks within PR regions. **d**, **h** Average profiles represent median PRO-seq intensity for the indicated samples around the +1 dyad defined using MNase-seq in the auxin-untreated Ino80p-AID cells (GSM3304635). **e** Boxplots indicate the relative read ratio (log_10_) at the significantly increasing peaks (the ratio of *arp5*Δ versus *wt*) in a manner similar to Fig. 3b (905 peaks for replicate 1 and 904 peaks for replicate 2 out of 1,346 peaks at 807 genes). **f** Boxplot depicts the distance from the +1 nucleosome dyad to the indicated pausing sites (bp) for shifted to 5’ genes in *arp5*Δ. **i** Overlap between shift-to-5’ genes upon Ino80p-KD and shifted to 5’ genes in *arp5*Δ. *P-value* was calculated using the hypergeometric distribution.

All PRO-seq data were generated using combined biological replicates. Only genes with nucleosomes overlapped with H3K4me3 ChIP-seq enrichment (GSM2507874) were used in an effort to exclude false-positive nucleosomes (260 no-shift genes upon Ino80p-KD, 118 inside genes and 142 outside genes; 406 not shifted genes in *arp5*Δ, 211 inside genes and 195 outside genes). For average profiles, medians reflect the 10-bp bin. Asterisks represent statistically significant differences, as calculated using either Wilcoxon Signed Rank Test or Mann-Whitney U Test.


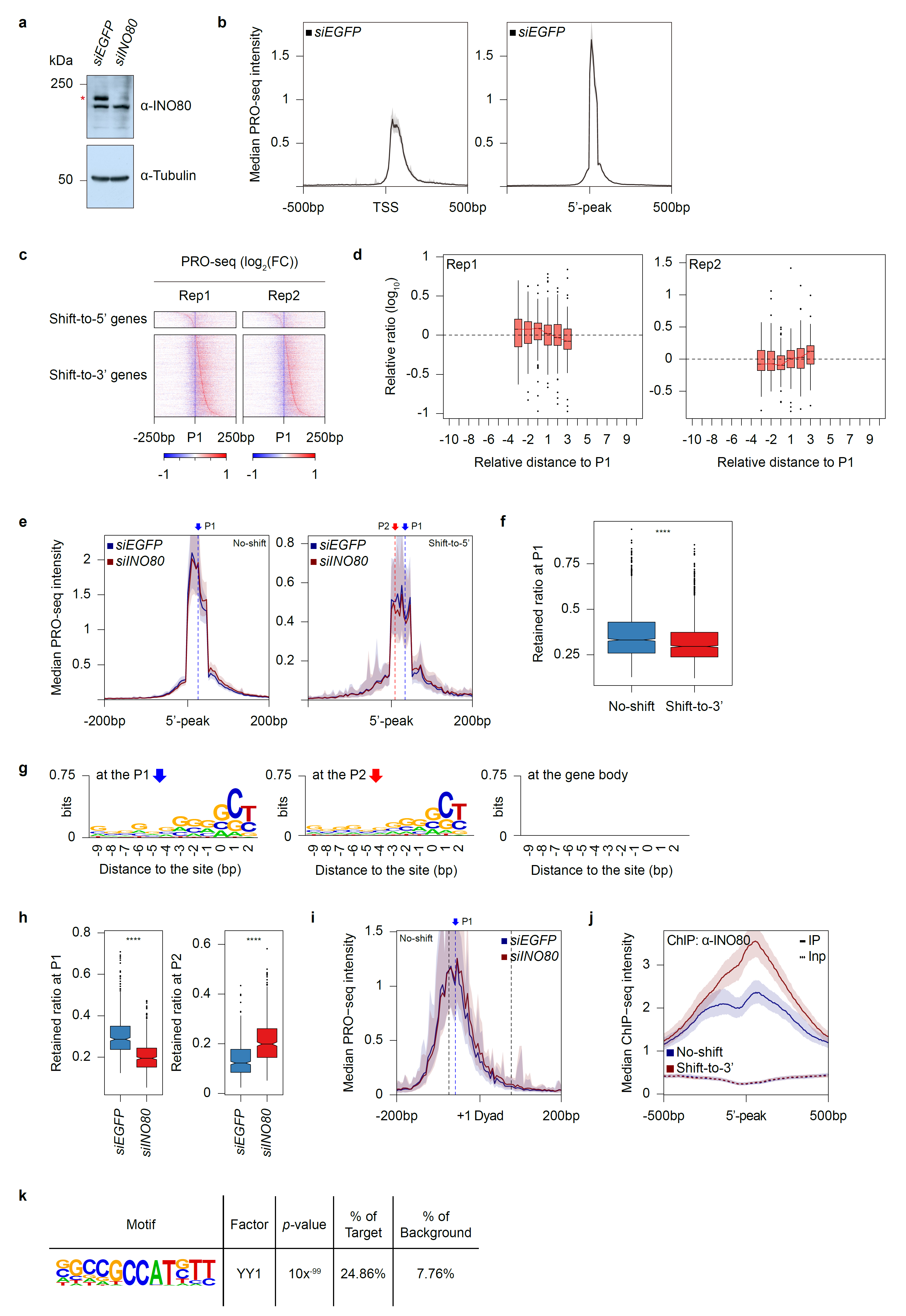


**Fig. S10. INO80 knockdown yields RNAPⅡ pausing site determination defect in mESCs.**

**a** Western blot analysis of whole-cell lysates from mESCs treated with *siEGFP* or *siINO80* for 48 hr showed that INO80 was almost completely and specifically degraded in the presence of *siINO80* (The red asterisk). Tubulin was detected as a loading control. **b** Average profiles show median PRO-seq intensity in *siEGFP*-treated mESCs centered on either annotated TSS or 5’-peak. **c** Heatmaps display the PRO-seq signal upon INO80-KD as a log_2_ fold change centered on P1 for each replicate. Genes were sorted by the distance from P1 to P2. **d** Boxplots represent the relative read ratio (log_10_) at the non-significantly increasing peaks (the ratio of *siINO80* versus those of *siEGFP*) in a manner similar to Fig. 3b (1,518 peaks for replicate 1 and 1,520 peaks for replicate 2 out of 1,835 peaks at 1,659 genes). **e** Average profiles show median PRO-seq intensity around 5’-peak of no-shift and shift-to-5’ genes. The dotted lines represent the median of P1 (blue) or P2 (red). **f**, **h** Boxplots indicate the ratio of smoothed PRO-seq intensity at the peaks corresponding to the indicated pausing sites to those sums of all peaks within PR regions. **g** Sequence logos at P1, P2, and the middle of the GB (gene body) regions of shift-to-3’ genes were generated using WebLogo [2]. **i** Average profile exhibits median PRO-seq intensity for the indicated samples around the +1 dyad for no-shift genes. Only genes with nucleosomes overlapped with H3K4me3 ChIP-seq enrichment (GSM590111) were used in an effort to exclude false-positive nucleosomes (N = 431). **j** Average profile displays median ChIP-seq intensity against INO80 in the J1 cell line mESCs (GSM1194194 and GSM1194195) at 5’-peak of no-shift and shift-to-3’ genes. **k** *De novo* Motif analysis using the findMotifsGenome.pl program of HOMER [4] found that the YY1 motif was significantly enriched at promoter regions (250 bp upstream and downstream of 5’-peak) of shift-to-3’ genes compared to no-shift genes.

All PRO-seq data were generated using combined biological replicates. For heatmaps, signals reflect the 1-bp bin. For average profiles, medians reflect the 10-bp bin. Asterisks represented statistically significant differences, as calculated using Mann-Whitney U Test.

| Experiment | Sample^a^ | Total reads | Mapped to rRNA | Uniquely mapped to genome | Spike-in genome | Spearman  correlation (ρ)^b^ | |
| --- | --- | --- | --- | --- | --- | --- | --- |
|  |  |  |  |  |  | PR | GB |
| PRO-seq | SC_Ino80p-AID_Ctrl_Rep1 | 26,142,062 | 11,900,913 | 5,501,869 | 322,452 | 0.986 | 0.995 |
|  | SC_Ino80p-AID_Ctrl_Rep2 | 25,041,456 | 11,493,690 | 5,282,796 | 322,219 |  |  |
|  | SC_Ino80p-AID_KD_Rep1 | 25,713,790 | 11,192,062 | 5,434,019 | 341,750 | 0.985 | 0.994 |
|  | SC_Ino80p-AID_KD_Rep2 | 22,761,013 | 10,326,913 | 4,624,242 | 324,736 |  |  |
|  | SC_Ino80p-AID_Rescue_Rep1 | 24,015,944 | 11,196,976 | 4,997,072 | 253,963 | 0.985 | 0.994 |
|  | SC_Ino80p-AID_Rescue_Rep2 | 28,013,177 | 13,288,181 | 5,407,738 | 275,995 |  |  |
| PRO-cap | SC_Ino80p-AID_Ctrl_Rep1 | 29,171,466 | 2,073,847 | 17,697,595 | 621,427 | 0.996 | N/A |
|  | SC_Ino80p-AID_Ctrl_Rep2 | 33,743,267 | 2,528,625 | 20,358,414 | 711,586 |  |  |
|  | SC_Ino80p-AID_KD_Rep1 | 27,699,506 | 1,537,081 | 18,037,675 | 783,854 | 0.995 |  |
|  | SC_Ino80p-AID_KD_Rep2 | 31,144,225 | 2,322,090 | 18,014,706 | 808,134 |  |  |
| PRO-seq | SC_*wt*_Rep1 | 25,155,762 | 8,671,835 | 6,399,398 | 399,563 | 0.987 | 0.994 |
|  | SC_*wt*_Rep2 | 26,429,266 | 10,004,941 | 6,435,134 | 351,368 |  |  |
|  | SC_*spt4*Δ_Rep1 | 21,261,858 | 2,758,684 | 11,162,014 | 321,189 | 0.996 | 0.983 |
|  | SC_*spt4*Δ_Rep2 | 22,331,041 | 4,475,130 | 11,536,938 | 302,405 |  |  |
|  | SC_*htz1*Δ_Rep1 | 24,700,582 | 10,071,645 | 5,543,700 | 287,164 | 0.986 | 0.994 |
|  | SC_*htz1*Δ_Rep2 | 29,874,171 | 11,196,944 | 7,006,309 | 355,166 |  |  |
|  | SC_*arp5*Δ_Rep1 | 23,312,472 | 7,394,892 | 6,670,498 | 394,804 | 0.988 | 0.996 |
|  | SC_*arp5*Δ_Rep2 | 23,135,952 | 7,778,575 | 6,603,702 | 340,276 |  |  |
|  | SP_ED665_Rep1 | 24,546,689 | 11,408,355 | 7,031,314 | 387,863 | 0.983 | 0.995 |
|  | SP_ED665_Rep2 | 27,273,312 | 12,477,181 | 7,762,793 | 428,951 |  |  |
|  | MM_E14Tg2a_*siEGFP*_Rep1 | 41,178,443 | 487,331 | 21,444,972 | N/A | 0.986 | 0.990 |
|  | MM_E14Tg2a_*siEGFP*_Rep2 | 60,330,200 | 750,424 | 33,427,705 |  |  |  |
|  | MM_E14Tg2a_*siINO80*_Rep1 | 60,798,641 | 732,917 | 33,253,699 |  | 0.988 | 0.991 |
|  | MM_E14Tg2a_ *siINO80*_Rep2 | 58,179,420 | 840,975 | 34,197,412 |  |  |  |

**Table S1. Summary of PRO-seq reads and reproducibility obtained in this study.**

^a^ SC indicates the *S. cerevisiae* sample, SP indicates the *S. pombe* sample, and MM indicates the mESCs sample. ^b^ Reproducibility of PRO-seq and PRO-cap was calculated by a Spearmans’ ρ at the indicated regions. For *S. cerevisiae*, the regions from observed TSS to downstream 250 bp (TSS to TSS +250 bp) were used as the promoter-proximal (PR) regions, and the regions from the downstream 250 bp to TES were used as the gene body (GB) regions. All filtered protein-coding genes (N = 5,697) were used for analysis. For *S. pombe*, the regions from TSS to downstream 100 bp were used as the PR regions, and the regions from the downstream 200 bp to TES were used as the GB regions. The gene set used in the previous study (N = 3,214) [1] was used for analysis. For mESCs, the regions from upstream 100 bp to downstream 200 bp of 5’-peak were used as the PR regions, and the regions from downstream 1 kb to TES were used as the GB regions. Filtered protein-coding genes considering only the isoform with the highest expression levels (N = 16,068) were used for analysis. For PRO-cap data, the regions 250 bp around the observed TSS (obsTSS -125 bp to obsTSS +125 bp) were used as the PR regions.

| # | Strain | Genotype | Reference |
| --- | --- | --- | --- |
| 1 | NF191 | INO80-IAA*-FLAG, genetic background:DF5^a^, paternal strain: U2721^b^, *his3-*Δ*200*, *leu2-3,2-112*, *lys2-801*, *trp1-1*(am), *URA3::TIR-9Myc*, *INO80-44AID9Flag::hphNT* | [3] |
| 2 |  | BY4741, genetic background S288C, paternal strain: BY4741, MATa *his3*Δ*1* *leu2*Δ*0* *met15*Δ*0* *ura3*Δ*0* |  |
| 3 | SC1264 | *spt4*Δ, genetic background S288C, paternal strain: BY4741, MATa *his3*Δ*1* *leu2*Δ*0* *met15*Δ*0* *ura3*Δ*0* *spt4*Δ*::natMX6* | This study |
| 4 | SC1266 | *htz1*Δ, genetic background S288C, paternal strain: BY4741, MATa *his3*Δ*1* *leu2*Δ*0* *met15*Δ*0* *ura3*Δ*0* *htz1*Δ*::natMX6* | This study |
| 5 | SC1086 | *arp5*Δ, genetic background S288C, paternal strain: BY4741, MATa *his3*Δ*1* *leu2*Δ*0* *met15*Δ*0* *ura3*Δ*0* *arp5*Δ*::kanMX6* | This study |

**Table S2. List of *S. cerevisiae* strains used in this study.**

Background and parental strain genotype are as follows. ^a^DF5: *MATa/MATα, lys2-801/lys2-801, leu2-3, 2-l12/leu2-3, 2-l12, ura3-52/ura3-52, his3-Δ200/his3-Δ200, trpl-l(am)/trpl-l(am)* [5]. ^b^U2721: *MATa, his3-Δ200, leu2-3,2-112, lys2-801, trp1-1(am), URA3::TIR-9Myc*.

**References**

1. Booth GT, Wang IX, Cheung VG, Lis JT. Divergence of a conserved elongation factor and transcription regulation in budding and fission yeast. Genome Res. 2016;26(6):799–811. https://doi.org/10.1101/gr.204578.116.

2. Crooks GE, Hon G, Chandonia JM, Brenner SE. WebLogo: a sequence logo generator. Genome Res. 2004;14(6):1188–90. https://doi.org/10.1101/gr.849004.

3. Klein-Brill A, Joseph-Strauss D, Appleboim A, Friedman N. Dynamics of chromatin and transcription during transient depletion of the RSC chromatin remodeling complex. Cell Rep. 2019;26:279–292.e275.

4. Heinz S, Benner C, Spann N, Bertolino E, Lin YC, Laslo P, et al. Simple combinations of lineage-determining transcription factors prime cis-regulatory elements required for macrophage and B cell identities. Mol Cell. 2010;38(4):576–89. https://doi.org/10.1016/j.molcel.2010.05.004.

5. Finley D, Özkaynak E, Varshavsky A. The yeast polyubiquitin gene is essential for resistance to high temperatures, starvation, and other stresses. Cell. 1987;48(6):1035–46. https://doi.org/10.1016/0092-8674(87)90711-2.
