# Supplementary material for: The chromatin remodeler Ino80 mediates RNAPII pausing site determination
Source: Genome Biol. 2021 Oct 18;22:294. doi: 10.1186/s13059-021-02500-1 (PMC8524862; doi:10.1186/s13059-021-02500-1)
Supplement: Supplementary file 2 — Additional file 2. Review history. [file 13059_2021_2500_MOESM2_ESM.docx]

**1^st^ round**

**Reviewer 1**

Previous research by several groups has shown the promoter associated RNA polymerase II pausing is a wide-spread feature of metazoan genes. In this paper by Cheon et al., use state-of-the-art approaches to precisely map pause sites and to show now that promoter-proximal pausing is also a wide-spread feature in budding and fission yeast, indicating that this is a conserved throughout eukaryotes. They show that the chromatin remodelling factor Ino80 regulates this pausing over many genes in yeast and in mammalian cells (embryonic stem cells), indicating a new role for this factor. This is linked to nucleosome position, apparently controlled by Ino80.

The data of this study are of high quality and convincing. The paper is in general well written (but see issues below). This is a nice study which will be of great interest to those studying promoter remodelling and RNA polymerase pausing.

The question that remains to me though is "What are the consequences of changing promoter proximal pause sites, e.g. by deleting or depleting Ino80?", "What is the link to gene expression and the biology of the cell"? I think the authors can address this by (primarily) performing gene ontology (and related) analysis of the genes that are affected and by examining aspects of gene expression. They could do this in their yeast system, in the ESCs or both. What can the authors say about the 476 genes that show a changed pausing pattern after Ino80 knockdown? Do these genes show any communality in terms of gene ontology, expression or changes in expression upon Ino80 knockdown? Is there any difference between the 'shift-to-5'' genes versus 'shift-to-3' genes?

What about gene ontology, function of the 1890 genes that showed a changed elongation peaks on Ino80 knockdown in ESC cells, especially the 'shift-to-3' genes? How does the Ino80 knockdown effect transcription in these cells and how is this linked to pausing?

Is any change in pausing sites linked to R-loop generation?

Additional issues:

1. The control budding yeast were actually grown in yeast peptone dextrose (YPD) containing ethanol (Ctrl). How does the growth in ethanol affect pausing compared to the normal growth condition, e.g. in rich glucose media without ethanol? I think the authors should state that in the text.

2. Figure 2d, How was PR density and pausing index calculated? I did not find this (maybe I missed it?). I found these figures hard to understand, maybe it could be explained better in the legend.

3. Page 11, line 12: where do the 350 'no-shift' genes suddenly come from? What is the link to P2? I think something went wrong there, like this sentence should be coming later.

4. Page 7, line 11: Biotin-11-CTP, not biotion-11-CTP.

5. The whole section from page 8, lines 37-55 ("Consistent with the previous results [9]…), I found hard to understand. I think the author should try to express more clearly here what they want to state. For example, line 54, what do they mean with 'rest group'? Could we find a more elegant way to describe 'not paused genes'? This could also be very confusing, as genes may not been paused simply because they are not transcribed much, or they do not show an increase binding of Pol II at the 5' end.

6. Page 11, lines 25-26: instead of "…rescue of Ino80p caused a tendency to restore this increase", I guess it should be "…to reset this increase" or even better "counteract this increase".

7. Page 11, lines 51-52: "..displaces..at locations" maybe better: "..relocates…to locations".

**Reviewer 2**

Cheon et al present a manuscript entitled "The chromatin remodeler Ino80 mediates RNAPII pausing site determination" wherein they document experiments using the PRO-seq methodology to examine RNAPII positioning in response to loss of Ino80, both in yeast S. cerevisiae and in mESCs. They describe altered RNAPII density in both systems but with distinct polarities. In budding yeast, there is an upstream shift towards the TSS from an observed position where RNAPII is found at increased density in WT cells. This position in WT is approximately between the +1 and +2 nucleosomes whereas the upstream shift moves into the +1 nucleosome. As Ino80 also controls nucleosome positioning, and there is increased fuzziness (less precise phasing) of nucleosomes upon loss of Ino80, a pause upstream is consistent with RNAPII potentially now pausing within a nucleosome that has altered position or has altered properties. In mESCs, there is a shift in subset of RNAPII downstream from normal pause position concomitant with loss of Ino80 shifting nucleosomes in general downstream. The manuscript title states that Ino80 mediates pause site determination and results are interpreted as differences between pausing "choice" upon loss of Ino80 rather than observed differences being due to altered relationship between RNAPII and nucleosome positioning or property. There are a number of framing choices that dilute the impact of the observations and the presentation might be improved in a number of ways.

General points:

1. The authors' observations of increased 5' density for RNAPII by PRO-seq methodology in budding yeast are counter to prior PRO-seq data from the Lis lab, but actually harmonize PRO-seq results with both NET-seq and ChIP-seq. The way this is presented is sort of haphazard and should be much more direct. First, reproducibility of any datasets here should be established in a set of figure panels (correlations between replicates for all experiments including pombe wt and spt4∆). This is not done until later figures and sort of tangentially. Second, it would be worthwhile to examine or demonstrate correlation between other published techniques as these results now unify our understanding of what might be happening at 5' ends in yeast.

2. It is critical that authors discuss limitations to PRO-seq in that only RNAPII that is able to run-on will be detected and any backtracked RNAPII will be invisible to the technique. This must be discussed up front as only a subset of RNAPII is actually detected. Additionally, the authors state that their PRO-seq is PRO-seq with minor modifications described by the Dynlacht lab. These modifications include the addition of ATP and GTP with biotinylated UTP and CTP. This means that some amount of precision is lost for RNAPII positioning. Kwak et al state that only a single biotinylated NTP can be added, however unbiotinylated NTPs (A,G) presumably can be added before and after, so the method employed is somewhat more akin to GRO-seq potentially. This would be a difference between the original Booth et al paper and here.

3. The figures are in general less accessible than they could be, including many things unlabeled or only discussed in legend, or labeled cryptically. For example, it is not useful to label figure with GSE accession numbers and not also what the datasets actually are. This makes thinking about the figures very difficult as will constantly need to go to the legend.

Other points.

Abstract

4. p. 2 l. 16-21 "In addition, these Ino80-dependent genes exhibit the +1 nucleosome that is closely associated with the alternative pausing site, which implied the suppression of the alternative pausing site in a nucleosome context dependent manner."

This is a difficult to parse sentence that centers the idea that Ino80 is suppressing a pause not that the pause is a result of altered Ino80 function. While these are two different ways of looking at the same data, it seems confusing to discuss Ino80 role as regulating a pause as opposed to promoting non-pausing.

5. p2 l. 26-28 "Moreover, we observed similar Ino80-dependent RNAPII pausing in mouse embryonic stem cells (mESCs)."

It is not clear to what extent the situations are similar. Being more precise - that pausing at 5' ends is altered when Ino80 is lost, though direction of effects and other aspects of pause regulation are different.

6. There are recent papers on both Ino80 mechanism https://rdcu.be/clzoP, https://rdcu.be/clzoS and RNAPII pausing in cerevisiae relating to a block between +1 and +2 nucleosomes https://www.biorxiv.org/content/10.1101/2021.03.03.433772v1.full that might be considered to be more sophisticated in the introduction. The introduction can also be much more direct that pausing will have different mechanisms in organisms based on factors present or absent, and that even in organisms where metazoan pausing apparatus is absent, there are data supporting regulation of elongation at 5' ends of genes. It is not clear if the link to prokaryotic pausing is relevant- regardless it should not be discussed as the 5' end of the lambda "gene"- lambda has many genes and promoters.

7. p4 l 8-10 "The most well-known function of Ino80 is to exchange the highly conserved histone variant H2A.Z for H2A [24, 25]"

It is not clear that Ino80 function is to exchange histones. It is important to be very precise and careful about references. Please see: https://science.sciencemag.org/content/353/6297/358.1.long

8. p4 line 16-19 "In addition, Ino80 is largely enriched at the TSS of most genes in yeast and mammals [25, 29], and has been shown to physically interact with the elongating RNAPII"

This physical interaction in the reference papers is described as recruitment to stalled RNAPII at DNA damage sites. It should not be presented as simple interaction with elongating RNAPII.

9. p4 l. 37-42, as in abstract, this framing is made difficult to parse.

10. p4 l. 43 "Further, we observed similar Ino80-dependent RNAPII pausing in mESCs"

Hard to say role is conserved when mechanism of pausing by definition has to be different in mammals. More precision would be welcome here.

Results

11. p4 l 52 "PRO-seq reveals genome-wide promoter-proximal pausing-like distributions in S. cerevisiae"

This result should be presented differently- this reconciles major differences between technologies and suggests that Pro-seq and NET-seq are much more similar for yeast than previously thought.

12. p5. l. 22-25 "Next, we classified genes as being paused or not paused based on promoter-proximal and gene-body mappable reads, as described in the previous study [17],.."

State here what the metric actual is, as this metric is very important for understanding figures. Also, x-y plots of pausing index in one dataset vs another might more easily allow evaluation than only looking at Venn diagrams. Reproducibility determination within own data should come first before comparison to any other data.

13. p5. l. 55 "Analysis of PROseq distribution for representative overlapped paused genes further showed that the accumulated PROseq signals within the promoter-proximal regions were not biased by the average profile (Fig. 1f).

Would like to get better idea of signal to noise- what about doing summation plot by decile or quintile based on control rank order? There are ways to visually analyze data in addition to just taking cutoffs.

14. p6. l. 17-21 "Consistent with the previous report [17], we found that spt4Δ caused the significant increase of PRO-seq intensity at the 5' end of the gene (Additional file 1: Fig. S1a, b; heatmaps were represented on the same scale in all of our data)"

Heat map not sufficient- how were heat maps normalized to sequencing depth and mapped reads- this should be made clear here to allow interpretation, not having to dig in methods. Where are reproducibility metrics for all the pombe data? Cannot be interpreted without clear discussion of the data generated.

15. p8 l. 52 "while the rest groups" -> missing word(s).

16. p9 l. 32 "(Fig. 1f)" should this be 2f?

17. p9 l. 41 "This difference suggested that RNAPII undergoes a longer elongation period before pausing in budding yeast compared to metazoans, and it could likely be attributed to the difference in promoter structure."

The obvious difference is the likely distinct mechanisms for pausing and this should be made explicit. The similarity can be discussed later if the idea is more clearly brought out that Ino80 might promote transit of a nucleosome- in both organisms the nucleosome could be the +1 as the +1 is upstream of putative pause window in yeast, and downstream of putative pause window in metazoans.

18. p9 l. 55. The putative role in elongation is not compelling because it is not a role in elongation that is described in the papers cited. It is a role in regulation of RNAPII after stall due to DNA damage.

19. p10 related to conditions of experiment. 3 hours would not necessarily be considered short or completely acute as most or all cells would have replicated or divided in this time frame. Just something to think about in terms of how to get as close as possible to direct Ino80 role.

20. p10 line 28-37. This description is not very easy to understand. I understand the challenge of finding metrics that allow you to do statistics based on changes at individual genes in addition to looking at median behavior. Think about how to make these descriptions more accessible.

21. p10 line 59 "For both gene sets, the increasing peak with the lowest p-value at each gene was selected…"

Perhaps this is fine, but it is confusing. What about alternatively taking one peak? Or median position within fit of smooth?

22. p11 line 41 "Surprisingly, we observed a distinct feature between two gene groups in that no-shift genes displayed a sharp and distinct peak whereas shift-to-5' genes"

I worry that the sharpness of peak on no shift genes is artifactual based on how the analysis is determining a peak and then aligning genes to that position.

23. p11 line 50-57. This is hard to understand. This framework is difficult (see above).

24. 11 line 52-57. "These results also implied that shift-to-5' genes bear a leakier P1 with more usage of an alternative pausing site than no shift genes, and may utilize Ino80p to facilitate RNAPII pausing at P1."

I have difficult making sense of this. Why is it concluded that Ino80 might be enforcing pausing at P1 rather than promoting overcoming of a barrier at P2?

25. p 12 l. 1-6 "…further suggesting the usage of P2 as an alternative pausing site and also the involvement of cis-acting nucleic acid sequences in RNAPII pausing."

This is perhaps too speculative. PRO-seq only detects ECs that can run on. Sequences here could be those that are less prone to backtracking if there is differential backtracking potential early in gene vs late and this might not be issue in rest of gene, hence differential enrichment.

26. p. 13. l. 13 "Surprisingly, the nucleosome distribution around P1 of shift-to-5' genes displayed a much better phase than that of no-shift genes (Fig. 4a, left)."

It would probably be useful to consider possible other correlating variables as expression or gene type/promoter class might be correlating as well. Trying to match for other variables between shift and no shift classes to then ask if difference is still apparent chould be useful.

27. p15 l. 28 "This suggested that Ino80-KD caused the transition of RNAPII solely toward locations where pausing also occurs but not a main pausing site."

This is unclear.

28. p15 l. 39 "Except for the direction of shift, however, Ino80-KD in mESCs yielded a pausing site determination defect similar to that observed in S. cerevisiae."

This is quite forced, "except for the direction of the shift, and the mechanisms of pausing being different..." If the analogy is instead that Ino80 has a role in transiting the +1 nucleosome, then the similarity is not as forced as it doesn't relate to mechanisms of pausing, it relates to where the +1 nucleosome is relative to the pause.

29. p16 l. 10-12. 1-4 "Based on our findings, we conclude that Ino80 plays a conserved role in determining where RNAPII should mainly pause at the genes with an alternative pausing site in the early transcription elongation stage in mESCs."

This seems strained. What if what is conserved is ino80 alters positions/dynamics of promoter adjacent nucleosomes and these in turn may affect pausing- especially in light of the analysis coming in the next paragraph?

30. p16 l "We observed the better phased nucleosome architecture around P1 for shift-to-3' genes relative to no-shift genes (Fig. 5e, up)."

Perhaps bootstrapping here to see if this analysis is robust to different ways to select subsets of promoters.

31. p16 line 26-30 "Consistently, we found that the ratio of P1 reads within promoter-proximal regions was significantly decreased upon Ino80-KD, whereas those of P2 showed a significant increase (Fig. 5g), further suggesting the significant increase of the promoter proximal RNAPII, which is accumulated at P2."

I don't understand this phrasing as these genes were identified already as having a shift?

32. p16 l. 40-44

Make it clear if these are same cell types or not. This is a simple but important result. Perhaps an additional experiment would be to ask if shift genes are enriched subset or not of Ino80 occupied 5' ends or not. Looking at correlation of Ino80 occupancy and shift or nucleosome characteristics from nuc-focused analysis. Do pausing differences emerge if you select promoters based on nuc data or Ino80 data or intersection of two? There is not really any analysis that attempts to define what these promoters are and what are the features that might be in common. It seems like the analysis could go deeper here just through examination of published data on mESCs. There are some simple analyses done on the yeast side as well.

Discussion

33. First line. I don't agree that the way the data are discussed indicates things are same between yeast and human- because discussion is focused on pausing at 5' end not the potential obstacle of +1 nucleosome, which is detected by altered distribution of PRO-seq reads from existing pauses that are based on different mechanisms toward new positions that may reflect inability to transit an Ino80-sensitive barrier.

34. p 17 line 22-26 "One of the reasons for this discrepancy might be that NELF, which is involved in the pausing of RNAPII at a more promoter-proximal region [54], is not conserved in yeasts, including S. cerevisiae and S. pombe [55, 56].

Both NELF and negative activity of DSIF. These ideas are already widely suggested in the literature so should be discussed differently.

35. p17 line 28 "The hypothesis is consistent with the PRO-seq distribution reported for S. pombe, which showed a near-overlapping association of RNAPII pausing with the +1 dyad [17]."

36. p17 line 34 language is awkward.

37. p17 line 37. There is no basis for "surprisingly" as mechanisms of pausing are different.

38. p18 line 28-35. An alternative is that Ino80 enables elongation through the P2 and is defective. It is difficult to say that relative reduction of P1 is happening or if an increase in P2 is from other populations that are releasing from P1 normally, either into what should be productive elongation or promoter proximal termination.

Conclusion

39. Reconsider reframing based on points above.

40. Please look over all figures for ways to improve figure labeling and figure legends, i.e. labeling of dashed lines on figures, annotation of datasets, schematics for metrics, etc

**Authors’ response**

Reviewer #1: Cheon et al. review

Previous research by several groups has shown the promoter associated RNA polymerase II pausing is a wide-spread feature of metazoan genes. In this paper by Cheon et al., use state-of-the-art approaches to precisely map pause sites and to show now that promoter-proximal pausing is also a wide-spread feature in budding and fission yeast, indicating that this is a conserved throughout eukaryotes. They show that the chromatin remodelling factor Ino80 regulates this pausing over many genes in yeast and in mammalian cells (embryonic stem cells), indicating a new role for this factor. This is linked to nucleosome position, apparently controlled by Ino80.

The data of this study are of high quality and convincing. The paper is in general well written (but see issues below). This is a nice study which will be of great interest to those studying promoter remodelling and RNA polymerase pausing.

**Reviewer’s comment:**

The question that remains to me though is "What are the consequences of changing promoter proximal pause sites, e.g. by deleting or depleting Ino80?", "What is the link to gene expression and the biology of the cell"? I think the authors can address this by (primarily) performing gene ontology (and related) analysis of the genes that are affected and by examining aspects of gene expression. They could do this in their yeast system, in the ESCs or both. What can the authors say about the 476 genes that show a changed pausing pattern after Ino80 knockdown? Do these genes show any communality in terms of gene ontology, expression or changes in expression upon Ino80 knockdown? Is there any difference between the 'shift-to-5'' genes versus 'shift-to-3' genes?

What about gene ontology, function of the 1890 genes that showed a changed elongation peaks on Ino80 knockdown in ESC cells, especially the 'shift-to-3' genes? How does the Ino80 knockdown effect transcription in these cells and how is this linked to pausing?

Is any change in pausing sites linked to R-loop generation?

**Our response:**

1) As pointed by the reviewer, it could be highly interesting to determine the consequences of changing pausing sites upon Ino80p-KD in terms of such as gene expression or gene ontology (GO). We have attempted to address this question and have examined that Ino80p-KD showed an increase or decrease patterns of gene body (GB) density (i.e., gene activity) reproducibly rather than a unidirectional change at 467 genes in *S. cerevisiae* (Rebuttal Fig. 1a). We have also not found the particular difference in GB density changes between shift-to-5’ or shift-to-3’ genes. We have encountered the problems in that PRO-seq results alone could not exactly interpret the changes in GB density. For example, a decrease in GB density could be attributed to the decrease in elongation processivity, increase in elongation rate or even increase in premature termination. The bidirectional changes in GB density have also been observed upon INO80-KD in 1,890 genes of mESCs (Rebuttal Fig. 1b). Nonetheless, one interesting point has been that shift-to-5’ genes in *S. cerevisiae* are highly enriched with the GO term related to nitrogen metabolism (Rebuttal Fig. 1c). We have not found any significantly enriched term in shift-to-3’ genes in budding yeast. In addition, we have observed that the top 10 significantly enriched terms in shift-to-3’ genes of mESCs included the terms related to nitrogen metabolism (Rebuttal Fig. 1d), suggesting that Ino80p regulates the pausing site of the conserved gene sets through budding yeast to mESCs. We have also not found any significantly enriched term in shift-to-5’ genes in mESCs. Previous studies have observed that the loss of Ino80 complex subunits caused alterations in nitrogen metabolism [1]. Thus, we would like to leave this question as a future study, such as what the consequences of a defect in pausing site determination upon Ino80p-KD in nitrogen stress are.
2) To address the link between R-loop generation and defect in pausing site determination, we analyzed the published DRIP-seq generated in E14 mESCs [2] (We could not find the DRIP-seq generated upon INO80 knockdown condition). When we compared the DRIP-seq signal at no-shift and shift-to-3’ genes in mESCs, we found that the DRIP-seq signal of shift-to-3’ genes was lower than those of no-shift genes at the downstream 1 kb of TSS. However, we found no significant difference at the immediate downstream of TSS. We also investigated the DRIP-seq signal in *S. cerevisiae* by analyzing the two different published data sets (SRP071346 [3] and GSE124132 [4]). However, we found that the distribution patterns were different at all published data in *S. cerevisiae*. Thus, we could not conclude whether these DRIP-seq patterns were reproducible and similar from budding yeast to mESCs. Therefore, we would like to exclude this part in the revised manuscript.

**Rebuttal Fig. 1.** **a**, **b** Heatmaps of the PRO-seq log_2_ fold change of GB density upon the loss of Ino80p in *S. cerevisiae* (**a**; 467 genes with increasing peaks) or mESCs (**b**; 1,890 genes with increasing peaks). **c**, **d** Gene ontology (GO) terms that are enriched in 353 shift-to-5’ genes in *S. cerevisiae* (**c**) or 1,658 shift-to-3’ genes in mESCs (**d**; the top 10 enriched terms based on the *p-value*). **e** Average profile of median DRIP-seq (GSM1720620) signal at 5’-peak of no-shift and shift-to-3’ genes in mESCs.

Additional issues:

**Reviewer’s comment:**

1. The control budding yeast were actually grown in yeast peptone dextrose (YPD) containing ethanol (Ctrl). How does the growth in ethanol affect pausing compared to the normal growth condition, e.g. in rich glucose media without ethanol? I think the authors should state that in the text.

**Our response:**

We would like to note that we added a tiny amount of ethanol (e.g.,100 μl of ethanol per 50 ml medium). In fact, PRO-seq data labeled as BY4741 in Fig. 1b was generated in YPD without ethanol. The heatmaps between Ctrl (generated in YPD containing ethanol) and BY4741 showed the striking similarity in distribution pattern around the TSS: the PR intensity of the two data correlated well (ρ = 0.9393586). Furthermore, the GB intensity of the two data also correlated well (ρ = 0.9606225). Thus, we concluded that the addition of ethanol does not affect the pausing pattern. According to the reviewer’s suggestion, we stated this point in the Results (p12) section. In order to make it easier to understand the issue of ethanol addition, the relevant part was stated immediately after the brief description of the AID system.

**Reviewer’s comment:**

2. Figure 2d, How was PR density and pausing index calculated? I did not find this (maybe I missed it?). I found these figures hard to understand, maybe it could be explained better in the legend.

**Our response:**

We are sorry that we did not explain how to get the density and calculation metric of the pausing index (PI) in detail. To be exact, PI was calculated as a ratio of PR density to GB density. PRO-seq density was calculated as the number of sense strand PRO-seq reads per mappable bases within the indicated region as described in the previous study [5, 6]. Mappable bases represented the bases uniquely aligned when aligning all possible 36-mer sequences of the genome to the same genome. As suggested by the reviewer, we explained these metrics in the Results (p6-7) and, in more detail, in the Methods (p28) section. Because the plot related to the PI was newly added in Fig. 1 due to the suggestion of Reviewer 2, the texts was added to the earlier part of the Results. (because of the word limit, we added this part in the main text rather than figure legends) (also related to Reviewer 2’s other point 12).

**Reviewer’s comment:**

3. Page 11, line 12: where do the 350 'no-shift' genes suddenly come from? What is the link to P2? I think something went wrong there, like this sentence should be coming later.

**Our response:**

We are sorry for the mistake. As pointed by the reviewer, that sentence should be came later. We revised the sentence as noted in the Results (p13) section.

**Reviewer’s comment:**

4. Page 7, line 11: Biotin-11-CTP, not biotion-11-CTP.

**Our response:**

We are sorry for our typo. We corrected it accordingly.

5. The whole section from page 8, lines 37-55 ("Consistent with the previous results [9]…), I found hard to understand. I think the author should try to express more clearly here what they want to state. For example, line 54, what do they mean with 'rest group'? Could we find a more elegant way to describe 'not paused genes'? This could also be very confusing, as genes may not been paused simply because they are not transcribed much, or they do not show an increase binding of Pol II at the 5' end.

**Our response:**

We would like to describe whether the promoter-proximal (PR) PRO-seq pattern in budding yeast is similar to those in metazoans or not: correlation between gene activity and PRO-seq PR density and inverse correlation between gene activity and PI. We observed these relationships for the paused genes in *S. cerevisiae* (Fig. 2d). However, when considering all genes, the group of genes with the lowest gene activity did not show a higher PI than the group of genes with the middle gene activity, resulting in not an inverse correlation between gene activity and PI (Additional file 1: Fig. S6b, right). In contrast, a previous study in human lung fibroblasts reported the inverse correlation between gene activity and PI when considering all genes also [6]. In the paragraph (p10-11), we tried to speculate the difference in this tendency. As we found the inverse correlation when considering paused genes in *S. cerevisiae* and also found that, when considering all genes, group of genes with middle gene activity showed the higher PI than those with highest gene activity, we simply implicated that the lower PI of the lowest gene activity group might be because these groups contain more not paused genes. Because not paused genes showed the lower PI than paused genes. In other words, gene activity of not paused genes in *S. cerevisiae* might be lower than generally in humans. We revised the paragraph more precisely in the Results (p10-11) section.

6. Page 11, lines 25-26: instead of "…rescue of Ino80p caused a tendency to restore this increase", I guess it should be "…to reset this increase" or even better "counteract this increase".

**Our response:**

Thank you for the suggestion. We revised the manuscript accordingly.

7. Page 11, lines 51-52: "..displaces..at locations" maybe better: "..relocates…to locations".

**Our response:**

Thank you for the suggestion. We revised the manuscript accordingly.

Reviewer #2: Cheon et al present a manuscript entitled "The chromatin remodeler Ino80 mediates RNAPII pausing site determination" wherein they document experiments using the PRO-seq methodology to examine RNAPII positioning in response to loss of Ino80, both in yeast S. cerevisiae and in mESCs. They describe altered RNAPII density in both systems but with distinct polarities. In budding yeast, there is an upstream shift towards the TSS from an observed position where RNAPII is found at increased density in WT cells. This position in WT is approximately between the +1 and +2 nucleosomes whereas the upstream shift moves into the +1 nucleosome. As Ino80 also controls nucleosome positioning, and there is increased fuzziness (less precise phasing) of nucleosomes upon loss of Ino80, a pause upstream is consistent with RNAPII potentially now pausing within a nucleosome that has altered position or has altered properties. In mESCs, there is a shift in subset of RNAPII downstream from normal pause position concomitant with loss of Ino80 shifting nucleosomes in general downstream.

**Reviewer’s comment:**

The manuscript title states that Ino80 mediates pause site determination and results are interpreted as differences between pausing "choice" upon loss of Ino80 rather than observed differences being due to altered relationship between RNAPII and nucleosome positioning or property. There are a number of framing choices that dilute the impact of the observations and the presentation might be improved in a number of ways.

**Our response:**

Given our key findings of the close association between the regulation of +1 nucleosome by Ino80p and the transition of pausing site upon the loss of Ino80p, our manuscript title is fair enough to state our central idea. Thus, we would like to leave the chance of using another framing in the manuscript title for a future study about the more detailed underlying mechanism.

General points:

**Reviewer’s comment:**

1. The authors' observations of increased 5' density for RNAPII by PRO-seq methodology in budding yeast are counter to prior PRO-seq data from the Lis lab, but actually harmonize PRO-seq results with both NET-seq and ChIP-seq. The way this is presented is sort of haphazard and should be much more direct. First, reproducibility of any datasets here should be established in a set of figure panels (correlations between replicates for all experiments including pombe wt and spt4∆). This is not done until later figures and sort of tangentially. Second, it would be worthwhile to examine or demonstrate correlation between other published techniques as these results now unify our understanding of what might be happening at 5' ends in yeast.

**Our response:**

1) We would like to note that Additional file: Table S1 included the summary of PRO-seq reads and reproducibility of all the replicates used in this study. We are sorry that we did not explain this part clearly in the manuscript. We added the relevant part in the Results (p6) section, where the reproducibility of replicates was first mentioned.
2) As suggested by the reviewer, we compared other published techniques, Rpb3 NET-seq and ChIP-exo, and found the positive correlation between them within both PR (ρ = 0.666218 between NET-seq and ChIP-exo_rep1 and ρ = 0.693254 between NET-seq and ChIP-exo_rep2) and GB (ρ = 0.767000 between NET-seq and ChIP-exo_rep1 and ρ = 0.766717 between NET-seq and ChIP-exo_rep2) regions (Fig. S5d). This helped us to more clearly unify the distribution pattern of early elongating RNAPⅡ in *S. cerevisiae*. We added the related part in the Results (p9) section.

**Reviewer’s comment:**

2. It is critical that authors discuss limitations to PRO-seq in that only RNAPII that is able to run-on will be detected and any backtracked RNAPII will be invisible to the technique. This must be discussed up front as only a subset of RNAPII is actually detected. Additionally, the authors state that their PRO-seq is PRO-seq with minor modifications described by the Dynlacht lab. These modifications include the addition of ATP and GTP with biotinylated UTP and CTP. This means that some amount of precision is lost for RNAPII positioning. Kwak et al state that only a single biotinylated NTP can be added, however unbiotinylated NTPs (A,G) presumably can be added before and after, so the method employed is somewhat more akin to GRO-seq potentially. This would be a difference between the original Booth et al paper and here.

**Our response:**

1) We agree that it is critical to describe the drawbacks of PRO-seq. We mentioned this part at the beginning of the Results (p6) section. Additionally, we explained the related part in the Introduction (p3 and 5) section more precisely.
2) We would like to note that the 2-Biotin run-on was initially described in the Mahat et al. (Nat. Protoc.) paper published by the Lis group [7] but not the Dynlacht lab. The Mahat et al. paper explained that “2-Biotin run-on provides reasonable resolution and cost” and the Kwak et al. paper showed that GRO-seq, the lower resolution approach of PRO-seq, agrees well with PRO-seq in general [8]. Based on these two points, we chose to perform the 2-Biotin run-on. Additionally, the Booth et al. paper mentioned that they used the 4-Biotin run-on instead of individual run-on techniques [5]. However, we agree that the use of 2-Biotin run-on leads to a decrease in the resolution and that it is important to describe the difference in the used techniques in detail. We are sorry for not including this part clearly. We added the related part in the Result (p7-8) section.

Reviewer’s comment:

3. The figures are in general less accessible than they could be, including many things unlabeled or only discussed in legend, or labeled cryptically. For example, it is not useful to label figure with GSE accession numbers and not also what the datasets actually are. This makes thinking about the figures very difficult as will constantly need to go to the legend.

**Our response:**

We agree with the reviewer on this point that some of the figures were less accessible. We labeled the figures more simply. For the previously published PRO-seq data, it was briefly indicated what the sample was below or above the sample accession number (GSM) when it was first displayed in the figure (Fig. 1, S1-3). For other previously published data, figures were labeled by the indicated methods or samples, and the detailed information of the sample accession number was indicated in the legend.

Other points.

Abstract

**Reviewer’s comment:**

4. p. 2 l. 16-21 "In addition, these Ino80-dependent genes exhibit the +1 nucleosome that is closely associated with the alternative pausing site, which implied the suppression of the alternative pausing site in a nucleosome context dependent manner."

This is a difficult to parse sentence that centers the idea that Ino80 is suppressing a pause not that the pause is a result of altered Ino80 function. While these are two different ways of looking at the same data, it seems confusing to discuss Ino80 role as regulating a pause as opposed to promoting non-pausing.

**Our response:**

We revised the Abstract section (p2) so that we explained our idea more clearly. One of our main results was that we observed the two different pausing sites, the main and the alternative one. We tried to describe that the role of Ino80p to regulate the +1 nucleosome is highly associated with the proper localization of RNAPII pausing at the main pausing site by suppressing RNAPII pausing at the alternative site. Additionally, we would like to note that PRO-seq signals at P2 in the Ctrl samples were above the basal levels (Fig. 3e, bottom), which is consistent with the hypothesis that RNAPII could also pause at P2 in the physiological state, but it does not represent a main pausing site. Thus, it is reasonable that pausing at P2 is not considered as non-pausing (also related to Reviewer 2’s other point 9).

**Reviewer’s comment:**

5. p2 l. 26-28 "Moreover, we observed similar Ino80-dependent RNAPII pausing in mouse embryonic stem cells (mESCs)."

It is not clear to what extent the situations are similar. Being more precise - that pausing at 5' ends is altered when Ino80 is lost, though direction of effects and other aspects of pause regulation are different.

**Our response:**

We revised that sentence more precisely (p2). We tone-downed and rewrote that INO80-KD causes a change in the pausing site instead of describing as simply that the role of Ino80p is conserved in mESCs. (also related to Reviewer 2’s other point 10).

**Reviewer’s comment:**

6. There are recent papers on both Ino80 mechanism https://rdcu.be/clzoP, https://rdcu.be/clzoS and RNAPII pausing in cerevisiae relating to a block between +1 and +2 nucleosomes https://www.biorxiv.org/content/10.1101/2021.03.03.433772v1.full that might be considered to be more sophisticated in the introduction. The introduction can also be much more direct that pausing will have different mechanisms in organisms based on factors present or absent, and that even in organisms where metazoan pausing apparatus is absent, there are data supporting regulation of elongation at 5' ends of genes. It is not clear if the link to prokaryotic pausing is relevant- regardless it should not be discussed as the 5' end of the lambda "gene"- lambda has many genes and promoters.

**Our response:**

We have thoroughly rewritten the Introduction section to be more detailed. In particular, the Introduction section was revised, focusing on the following three parts: First, the drawbacks of PRO-seq that it cannot detect a non-elongation-competent form of RNAPⅡ. Second, past studies suggesting that pausing-like features are conserved in *S. cerevisiae* that lacks NELF homologous. Third, a more elaborate explanation of the remodeling activity of the Ino80p (also related to Reviewer 2’s other points 7 and 8), especially in the point of the histone dimer exchange. In addition, we deleted the content related to pausing shown in the *E. coli* lambda gene, as we agree with the reviewer on this point that there has not been the exact link between pausing in prokaryotes and those in eukaryotes. This also helped us to integrate and discuss the previous literature that we have overlooked in the previous manuscript more appropriately.

**Reviewer’s comment:**

7. p4 l 8-10 "The most well-known function of Ino80 is to exchange the highly conserved histone variant H2A.Z for H2A [24, 25]"

It is not clear that Ino80 function is to exchange histones. It is important to be very precise and careful about references. Please see: <https://science.sciencemag.org/content/353/6297/358.1.long>

**Our response:**

We agree with the reviewer that the role of the Ino80p in histone exchange should be dealt with more precisely based on the previous literature. We added not only the technical comment mentioned by the reviewer and but also the related previous literatures that were written by the Wu [9, 10] and the Peterson group [11, 12].

**Reviewer’s comment:**

8. p4 line 16-19 "In addition, Ino80 is largely enriched at the TSS of most genes in yeast and mammals [25, 29], and has been shown to physically interact with the elongating RNAPII"

This physical interaction in the reference papers is described as recruitment to stalled RNAPII at DNA damage sites. It should not be presented as simple interaction with elongating RNAPII.

**Our response:**

We agree that we have overlooked this point in the previous manuscript. Also, we agree that there was a problem with the citation of the Poli et al. paper [13] and discarded it in the references. However, we would like to note that the Lafon et al. paper [14] mentioned that “*Interestingly, Rpb1 was identified in our proteomic screen and in a co-IP assay for Ino80-TAP (data not shown and Figure 5A). Both Ino80 and Arp5 associated with Rpb1 in a reciprocal co-IP against Rpb1-GFP, confirming the interaction between INO80 and RNAPII (Figure 5B). Furthermore, Rpb1S2P co-precipitated with Arp5 in a co-IP assay (**[Figure 5](https://www.sciencedirect.com/science/article/pii/S1097276515008199?via%3Dihub#fig5)C). This result implies that INO80 is in contact with elongating RNAPII.*”. Thus, we cited this paper as the Ino80 complex has been suggested to physically interact with the pSer2 form of elongating RNAPⅡ.

**Reviewer’s comment:**

9. p4 l. 37-42, as in abstract, this framing is made difficult to parse.

**Our response:**

We discussed this part in detail in Reviewer 2’s other point 4, which we described above. We revised the Abstract sections accordingly.

**Reviewer’s comment:**

10. p4 l. 43 "Further, we observed similar Ino80-dependent RNAPII pausing in mESCs"

Hard to say role is conserved when mechanism of pausing by definition has to be different in mammals. More precision would be welcome here.

**Our response:**

We discussed this part in Reviewer 2’s other point 5, which we described above. We revised the Introduction (p4) sections.

Results

**Reviewer’s comment:**

11. p4 l 52 "PRO-seq reveals genome-wide promoter-proximal pausing-like distributions in S. cerevisiae"

This result should be presented differently- this reconciles major differences between technologies and suggests that Pro-seq and NET-seq are much more similar for yeast than previously thought.

**Our response:**

We would like to note that we have tried to describe how the similarity between the different technologies that could detect the elongation complexes through the entire paragraph, especially in the Fig. S5. We also postulated the plausible reason underlying the difference between the previous data and our data, and it might lead to a strong correlation between PRO-seq and NET-seq, which was not observed in the previous report [5]. We added the description that the similarity between the two different technologies was much stronger than previously thought in the Results (p9) section. We also revised the subheading to imply that the promoter-proximal peak of RNAPⅡ could be observed in *S. cerevisiae* regardless of the type of technology that can detect the elongation complexes.

**Reviewer’s comment:**

12. p5. l. 22-25 "Next, we classified genes as being paused or not paused based on promoter-proximal and gene-body mappable reads, as described in the previous study [17],.."

State here what the metric actual is, as this metric is very important for understanding figures. Also, x-y plots of pausing index in one dataset vs another might more easily allow evaluation than only looking at Venn diagrams. Reproducibility determination within own data should come first before comparison to any other data.

**Our response:**

1) We stated the calculation metric of PI in detail in Reviewer 1’s additional issue 2, which we described above in the Results (p6-7) section.
2) We generated the scatter plot of PI between the two different data and found a positive correlation. We added these scatter plots for all the compared PRO-seq data sets (Fig. 1c, S1b, S2b, S3b).
3) We discussed the reproducibility of all the PRO-seq data generated in this study in detail in Reviewer 2’s general point 1, which we described above.

**Reviewer’s comment:**

13. p5. l. 55 "Analysis of PROseq distribution for representative overlapped paused genes further showed that the accumulated PROseq signals within the promoter-proximal regions were not biased by the average profile (Fig. 1f).

Would like to get better idea of signal to noise- what about doing summation plot by decile or quintile based on control rank order? There are ways to visually analyze data in addition to just taking cutoffs.

**Our response:**

As suggested by the reviewer, we first binned genes in quartiles based on the PI of our PRO-seq data or the published PRO-seq data. We then compared the average profile of each quartile and found that the PI of the two different data was highly related. It was consistent with the positive value of Spearman’s ρ between the two data. We performed these analyzes based on PI quartiles not only on *S. cerevisiae* (Fig. 1d, e) but also on *S. pombe* (Fig. S2c, d) and mESCs (Fig. S3c, d) data and added them in the revised manuscript appropriately.

**Reviewer’s comment:**

14. p6. l. 17-21 "Consistent with the previous report [17], we found that spt4Δ caused the significant increase of PRO-seq intensity at the 5' end of the gene (Additional file 1: Fig. S1a, b; heatmaps were represented on the same scale in all of our data)"

Heat map not sufficient- how were heat maps normalized to sequencing depth and mapped reads- this should be made clear here to allow interpretation, not having to dig in methods. Where are reproducibility metrics for all the pombe data? Cannot be interpreted without clear discussion of the data generated.

**Our response:**

1) We stated how we normalize the PRO-seq data in the up-front of the Results (p6) section. Since, in this initial part of the Results section, two separately generated data sets were compared, the experimental reads were normalized to reads per million mapped reads (RPM) without considering spike-in counts. However, we agree with the reviewer that heatmap is not sufficient to compare the changes in PR intensity. Thus, we compared the PI between *wt* and *spt4*Δ and found that *spt4*Δ caused a significant increase in the PI as in the previous study [5] (Fig S1c, d).
2) In the downstream part of the Results section, where we compared the PRO-seq data of Ctrl and KD or *wt* and *arp5*Δ, we normalized the experimental reads to the relative number of spike-in counts, and these normalized reads were presented in RPM considering the sequencing depth of the experimental reads. We stated this normalization factor in the Results (p12) section. We recognize that this normalization method was different from the method used by the Booth et al. paper [5, 15]. The Booth et al. paper normalized the experimental reads only to the relative number of spike-in counts [5] or the total spike-in mapped reads/10^5^ [15]. However, we considered the sequencing depth, because in some instances, such as *spt4*Δ, experimental reads increased significantly (Table S1).
3) Because we identified the increasing P2 based on the changes in the read ratio of the peak to P1, we were able to identify peaks almost independent of the read normalization method. Furthermore, we additionally analyzed the data normalized to either the relative number of spike-in counts only as in the Booth et al. paper (2016) [5] or the total spike-in mapped reads/10^5^ as in the Booth et al. paper (2018) [15], and examined the strikingly similar results in both cases. We successfully identified the almost similar shift-to-5’ genes with increasing upstream P2: 352 genes by the 2016 normalization factor and 351 genes by the 2018 normalization factor (and both entirely overlap with the 353 genes we first identified). In our data, the only thing that could be affected by the normalization factor is Fig. 3e, which showed the difference between the peaks at P1 and P2, so we checked whether this is reproduced when we used the other normalization factors. As a result, we still found the significant decrease of PRO-seq signal at P1 and the increase at P2 upon the loss of Ino80p accompanied by the opposite tendency upon the subsequent rescued conditions (Rebuttal Fig. 2). Thus, we conclude that the increasing upstream P2 upon Ino80p-KD was shown in our data was not due to an analytical bias.
4) We initially used the *S. pombe* PRO-seq data that was not generated as biological duplicates. We conducted a re-analysis using data generated from biological replicas and confirmed patterns strikingly similar to published data (Fig. S2). As we described above, the reproducibility of all the PRO-seq data used in this study was presented in Table S1.

**Rebuttal Fig. 2**. Average profiles of median PRO-seq intensity and boxplots of the smoothed PRO-seq intensity at the indicated pausing sites of shift-to-5’ genes that were calculated by the 2016 normalization factor [5] (**a**) or the 2018 normalization factor [15] (**b**).

**Reviewer’s comment:**

15. p8 l. 52 "while the rest groups" -> missing word(s).

**Our response:**

We are sorry for our typo. We revised the text precisely to indicate what the rest groups are.

**Reviewer’s comment:**

16. p9 l. 32 "(Fig. 1f)" should this be 2f?

**Our response:**

We are sorry for our mistake. We corrected it accordingly.

**Reviewer’s comment:**

17. p9 l. 41 "This difference suggested that RNAPII undergoes a longer elongation period before pausing in budding yeast compared to metazoans, and it could likely be attributed to the difference in promoter structure."

The obvious difference is the likely distinct mechanisms for pausing and this should be made explicit. The similarity can be discussed later if the idea is more clearly brought out that Ino80 might promote transit of a nucleosome- in both organisms the nucleosome could be the +1 as the +1 is upstream of putative pause window in yeast, and downstream of putative pause window in metazoans.

**Our response:**

We deleted the related texts in the Results (p12) sections. Rather, we added the speculation about the plausible reason of the distinct aspects of RNAPⅡ pausing-like features in *S. cerevisiae* in the Discussion (p20-21) sections in detail.

**Reviewer’s comment:**

18. p9 l. 55. The putative role in elongation is not compelling because it is not a role in elongation that is described in the papers cited. It is a role in regulation of RNAPII after stall due to DNA damage.

**Our response:**

We revised the manuscript to state that the role of Ino80p in transcriptional regulation, not elongation, was evident.

**Reviewer’s comment:**

19. p10 related to conditions of experiment. 3 hours would not necessarily be considered short or completely acute as most or all cells would have replicated or divided in this time frame. Just something to think about in terms of how to get as close as possible to direct Ino80 role.

**Our response:**

We used the 3 hr time point for a knockdown to make sure the depletion of Ino80p. Furthermore, we believe that the defect in pausing site determination upon Ino80p-KD in our study was Ino80p-dependent phenotype, as we also observed that the rescued Ino80p counteract the defect shown in KD samples.

**Reviewer’s comment:**

20. p10 line 28-37. This description is not very easy to understand. I understand the challenge of finding metrics that allow you to do statistics based on changes at individual genes in addition to looking at median behavior. Think about how to make these descriptions more accessible.

**Our response:**

The detailed description of our analysis method is as follows. We first smoothed each PRO-seq data with a Savitzky-Golay smoothing method [38] using the R sgolayfilt function (p = 4 and n = 7) to increase the accuracy and precision without distorting the PRO-seq signal. For each gene, we then identified consensus peaks present across all samples in the data set. During this process, we filtered out non-systematic peaks with 3-folds lower than the mean value of PR signals. We calculated the smoothed read count ratios of all the other peaks to P1. For each gene, we calculated t-statistic values for the observed ratios of peaks in the samples between two conditions. We also generated an empirical null distribution for t-statistic values by calculating t-statistic values after randomly permuting the read count matrix 1,000 times. The adjusted p-value was calculated by performing the left-sided test for its observed read count ratio using the empirical distribution. Finally, we selected the increasing peaks as the ones with a *p-value* < 0.1. We further filtered out false-positive peaks with log_2_ median ratios lower than a cutoff after estimating an empirical null distribution of log_2_ median ratios for the smoothed read count ratios between the two conditions during the above random permutations. The cutoff was determined as the mean of 10^th^ and 90^th^ percentiles in the empirical distribution. However, it was too complicated to describe all of this in the Results section, so we briefly added how to define the consensus peak and obtain the read ratio for P1 of each peak in the Results (p12-13) section. We hope the revised description in the Results section would be more accessible.

**Reviewer’s comment:**

21. p10 line 59 "For both gene sets, the increasing peak with the lowest p-value at each gene was selected…"

Perhaps this is fine, but it is confusing. What about alternatively taking one peak? Or median position within fit of smooth?

**Our comment:**

First of all, we would like to note that the genes with multiple P2 were not so many. Only 77 genes out of 353 genes of shift-to-5’ genes showed multiple P2 in *S. cerevisiae* (Rebuttal Fig. 3a), and 545 genes out of 1658 genes showed multiple P2 in mESCs (Rebuttal Fig. 3c). Furthermore, most of them had only two P2s, not many P2s. Also, if the number of groups differs, statistical analysis could be biased, so we would like to select one peak that was most affected per gene in order to proceed with the paired test. Thus, we determined to define the peak with the lowest *p-value* as P2. However, we also analyzed the position of not selected increasing peaks. When we considered all increasing peaks or randomly selected the increasing peak per gene, we still observed that most of them were positioned close to the +1 dyad compared to P1 (Rebuttal Fig. 3b, d). Collectively, we believe that it was reasonable to identify the peak with the lowest *p-value* as P2. Additionally, since we would like to define the precise position of P2, we thought that the latter case, median position within the fit of smooth, was not suitable for our analysis.

**Rebuttal Fig. 3.** **a**, **c** Histograms of the number multiple P2 for the genes with multiple P2 in *S. cerevisiae* (**a**) or mESCs (**c**). **b**, **d** Boxplots of the distance from the +1 dyad to the indicated pausing sites of each shifted gene set in *S. cerevisiae* (**b**) or mESCs (**d**). Both cases of all increasing peaks (i.e., all P2) or randomly selected the increasing peak per gene (i.e., random P2) were considered.

**Reviewer’s comment:**

22. p11 line 41 "Surprisingly, we observed a distinct feature between two gene groups in that no-shift genes displayed a sharp and distinct peak whereas shift-to-5' genes"

I worry that the sharpness of peak on no shift genes is artifactual based on how the analysis is determining a peak and then aligning genes to that position.

**Our response:**

We agree that the sharpness of the peak shown in the average profile could be affected depending on where it is aligned. In order to quantify the use of P1 at the two different gene sets, we calculated the retained ratio at P1 of no-shift and shift-to-5’ genes for each gene. The retained ratio at P1 was calculated by the ratio of the PRO-seq signal at P1 to those at all other consensus peaks within PR regions. Indeed, we found that the retained ratio at P1 of no-shift genes was significantly higher than those of shifted genes in both *S. cerevisiae* and mESCs (Fig. S8d, S10f). This difference in usage of P1 also showed in the representative genes (Rebuttal Fig. 4). Thus, we conclude that P1 of shifted genes was leakier than those of no-shift genes in *S. cerevisiae* and mESCs. We added the boxplot of retained ratio at P1 of both gene sets and the relevant description in the Results (p14 and 18-19) section (also related to Reviewer 2’s other point 23).

**Rebuttal Fig. 4.** Genome browser view of PRO-seq signal for representative shift-to-5’ genes (**a**) or no-shift genes (**b**) in *S. cerevisiae* and representative shift-to-3’ genes (**c**) or no-shift genes (**d**) in mESCs.

**Reviewer’s comment:**

23. p11 line 50-57. This is hard to understand. This framework is difficult (see above).

**Our response:**

As discussed in the above comment (Reviewer 2’s other comment 22), we quantified that the leakiness of P1 was significantly different at the two different gene sets, no-shift and shift-to-5’ genes (Rebuttal Fig. 3). We also revised the manuscript more precisely in the Results (p14) sections.

**Reviewer’s comment:**

24. 11 line 52-57. "These results also implied that shift-to-5' genes bear a leakier P1 with more usage of an alternative pausing site than no shift genes, and may utilize Ino80p to facilitate RNAPII pausing at P1."

I have difficult making sense of this. Why is it concluded that Ino80 might be enforcing pausing at P1 rather than promoting overcoming of a barrier at P2?

**Our response:**

We agree that the data shown in Fig. 3d alone is difficult to distinguish the two possibilities mentioned by the reviewer, facilitating pausing at P1 and overcoming a barrier at P2. Therefore, we deleted the last part of the sentence to speculate the mechanism after dealing with more data in the Results (p14-15) section.

**Reviewer’s comment:**

25. p 12 l. 1-6 "…further suggesting the usage of P2 as an alternative pausing site and also the involvement of cis-acting nucleic acid sequences in RNAPII pausing."

This is perhaps too speculative. PRO-seq only detects ECs that can run on. Sequences here could be those that are less prone to backtracking if there is differential backtracking potential early in gene vs late and this might not be issue in rest of gene, hence differential enrichment.

**Our response:**

We would like to note that gene body regions used in the Fig. S8e (and also in the Fig. S10g) were not a pausing site. This region was used as a negative control to examine whether the sequence preference at P1 and P2 was specific for the pausing site but not artifactual bias by the biotin incorporation.

**Reviewer’s comment:**

26. p. 13. l. 13 "Surprisingly, the nucleosome distribution around P1 of shift-to-5' genes displayed a much better phase than that of no-shift genes (Fig. 4a, left)."

It would probably be useful to consider possible other correlating variables as expression or gene type/promoter class might be correlating as well. Trying to match for other variables between shift and no shift classes to then ask if difference is still apparent could be useful.

**Our response:**

We would like to note that we have also tried to figure out the common feature of these shifted genes. We have compared the gene activity in Ctrl samples, shown in GB density, at the two different gene sets (Rebuttal Fig. 5a, d). In addition, we have compared the gene length or the distance to the closest upstream TSS and found no significant changes (Rebuttal Fig. 5b, e and c, f) in both *S. cerevisiae* and mESCs. We have further searched whether a specific chromatin-associated factor was highly enriched in the promoter of shifted genes based on the published ChIP-exo data in *S. cerevisiae* (GSE147927; 63 factors including Abd1p, Acs1p, Adk1p, Ado1p, Apc9p, Apt1p, Arp9p, Bat2p, Bre2p, Cdd1p, Dcc1p, Dus1p, Elp3p, Erg8p, Esc8p, Fap1p, Fsh1p, Glc7p, Gre2p, Gre3p, Grx3p, Grx4p, Hor2p, Hst2p, Ids2p, Lot5p, Mor1p, Mxr1p, Nto1p, Oaf1p, Pab1p, Plp2p, Rad2p, Rai1p, Rib4p, Rmt2p, Rsc2p, Sch9p, Sgi1p, Slt2p, Std1p, Thp1p, Utp4p, Yhm2p, Arg80p, Bud20p, Bud32p, Cdc33p, Cdc48p, Ecm15p, Rtf1p, Yra1p, Ysa1p, Ypr1p, Gal83p, Hst4p, Kap95p, Mre11p, Nic96p, Rad52p, Rad57p, Taf11p, Tip41p) [16]. However, unfortunately, we did not find the specific features at the promoter regions of these Ino80p-dependent shifted genes. Thus, we speculated that the nucleosome distribution pattern around P1 was critical for pausing site determination at these genes (Fig. 4a). It seemed that the +1 nucleosome dyad of shifted genes is located at more constant positions than no-shift genes around P1, resulting in much better-phased distribution around P1. Based on these results, we hypothesized that the nucleosome context itself is strongly associated with alternative pausing sites, and cells might employ the nucleosome context to suppress the RNAPⅡ pausing at the alternative pausing site, thereby establishing RNAPⅡ pausing at the main pausing site. Ino80p seems to be critical in this process through its role in regulating the +1 nucleosome.

**Rebuttal Fig. 5.** Boxplots of PRO-seq GB density (**a**, **d**), gene lengths (**b**, **e**), and the distance to the closest upstream TSS (**c**, **f**) at the indicated gene set. **a**-**c** for *S. cerevisiae* data and **d**-**f** for mESCs data.

**Reviewer’s comment:**

27. p15 l. 28 "This suggested that Ino80-KD caused the transition of RNAPII solely toward locations where pausing also occurs but not a main pausing site."

This is unclear.

**Our response:**

Fig. 5c showed the PRO-seq signal in *siEGFP*-treated samples at P1 of no-shift and shift-to-3’ genes. We found that the distribution pattern of the two signals was slightly different in that shift-to-5’ genes presented the small accumulation of PRO-seq signals downstream of P1. It is interesting that this accumulation occurred at the locations where the majority of P2 was located. Thus, we speculated that Ino80p-KD relocates RNAPⅡ particularly to locations where pausing also occurs but not a main pausing site. We revised that paragraph (p18-19) to be more detailed so that what we tried to describe would be more accessible. We also added the label on the dotted lines of the average plot to make it easier to understand.

**Reviewer’s comment:**

28. p15 l. 39 "Except for the direction of shift, however, Ino80-KD in mESCs yielded a pausing site determination defect similar to that observed in S. cerevisiae."

This is quite forced, "except for the direction of the shift, and the mechanisms of pausing being different..." If the analogy is instead that Ino80 has a role in transiting the +1 nucleosome, then the similarity is not as forced as it doesn't relate to mechanisms of pausing, it relates to where the +1 nucleosome is relative to the pause.

**Our response:**

Because the analysis and description of the association of RNAPⅡ pausing with the +1 nucleosome were focused on the next paragraph, we deleted the texts in this part (p18). We would like to focus here that the INO80-dependent genes in mESCs also presented an alternative pausing site as observed in *S. cerevisiae*. We revised the manuscript as described, rather than simply mentioning the similarity between a defect in pausing site determination in the two different organisms.

29. p16 l. 10-12. 1-4 "Based on our findings, we conclude that Ino80 plays a conserved role in determining where RNAPII should mainly pause at the genes with an alternative pausing site in the early transcription elongation stage in mESCs."

This seems strained. What if what is conserved is ino80 alters positions/dynamics of promoter adjacent nucleosomes and these in turn may affect pausing- especially in light of the analysis coming in the next paragraph?

**Our response:**

We agree that it was hasty to use the word "conclude" before the relationship to the nucleosome was even described. We deleted the sentence and revised the sentence at the end of the Results (p20) section to propose our idea in tone-downed state.

30. p16 l "We observed the better phased nucleosome architecture around P1 for shift-to-3' genes relative to no-shift genes (Fig. 5e, up)."

Perhaps bootstrapping here to see if this analysis is robust to different ways to select subsets of promoters.

**Our response:**

As suggested by the reviewer, we analyzed the nucleosome distribution based on the bootstrapped estimation. We randomly generate 1,000 bootstrapped samples. We calculated the median and confidence intervals using each average of subsamples. We revised the figures (Fig. 4a, 5e) and added the related description in the Results (p16, 19) section.

**Reviewer’s comment:**

31. p16 line 26-30 "Consistently, we found that the ratio of P1 reads within promoter-proximal regions was significantly decreased upon Ino80-KD, whereas those of P2 showed a significant increase (Fig. 5g), further suggesting the significant increase of the promoter proximal RNAPII, which is accumulated at P2."

I don't understand this phrasing as these genes were identified already as having a shift?

**Our response:**

We would like to note that, as described in the and the Results and Methods section in detail, we identified the increasing peaks using the ratio of reads with respect to P1 rather than using reads at peak. We would like to examine the relative enrichment pattern of RNAPⅡ between the main and alternative pausing sites. Thus, we decided that the comparison of the read ratio was more important than the comparison of the reads themselves. This analysis method has the advantage of being able to exclude the case where the P1 reads also increased. Therefore, we would like to note that because P2 is selected using the reads ratio, examining the changes in reads at P1 and P2 was not a repetitive analysis, but a meaningful analysis. We also discussed this part in the above comment (Reviewer 2’s other comment 20), and briefly added how to obtain the read ratio for P1 of each peak in the Results (p12-13) section.

**Reviewer’s comment:**

32. p16 l. 40-44

Make it clear if these are same cell types or not. This is a simple but important result. Perhaps an additional experiment would be to ask if shift genes are enriched subset or not of Ino80 occupied 5' ends or not. Looking at correlation of Ino80 occupancy and shift or nucleosome characteristics from nuc-focused analysis. Do pausing differences emerge if you select promoters based on nuc data or Ino80 data or intersection of two? There is not really any analysis that attempts to define what these promoters are and what are the features that might be in common. It seems like the analysis could go deeper here just through examination of published data on mESCs. There are some simple analyses done on the yeast side as well.

**Our response:**

1) We added the cell line information of that ChIP-seq data [17] in the Results (p19) section. The ChIP-seq data generated in the J1 cell line of mESCs.
2) We would like to note that Fig. S9a displayed no significant difference in chromatin localized enrichment of Ino80p calculated by ChEC-seq [18] between no-shift and shift-to-5’ genes in *S. cerevisiae*. As we described in Reviewer 2’s other point 26, we speculated that the nucleosome distribution pattern around P1 itself might be essential in pausing site determination at the Ino80p-dependent genes in *S. cerevisiae*. We also postulated that the high enrichment of mammalian INO80, which was a distinct result as in *S. cerevisiae*, could be the presence of mammalian INO80 specific subunit YY1 in the Results (p20) section.
3) In addition, we analyzed the correlation between Ino80p occupancy and changes in nucleosome distribution. We used the ChEC-seq signal at the TSS that the Kubik et al. paper presented in the supplementary table [18], as Ino80p occupancy. We calculated the nucleosome changes upon Ino80p-KD that were detected closest to the Ino80p signal by Danpos2 [19]. We found almost no correlation between the Ino80p ChEC-seq signal and changes in the nucleosome fuzziness (Rebuttal Fig. 6a). Accordingly, when we binned the genes in quartiles based on the changes in nucleosome fuzziness, we observed that Ino80p occupancy does not necessarily point to changes in nucleosome fuzziness (Rebuttal Fig. 6b). Thus, it is reasonable that the selection of genes based on the changes in nucleosome fuzziness or Ino80p enrichment was not suitable in analyzing the transition in RNAPⅡ pausing site. We would like to note that we did not analyze the nucleosome distribution upon the loss of INO80 in mESCs (we could not find the related published data on mESCs) and added the description of this part in the Discussion (p22) sections. Thus, we would like to leave the question about mESCs part for the future study.

**Rebuttal Fig. 6.** **a** Scatterplot of the correlation of ChEC signal and changes in the fuzziness of nucleosome. The ChEC-seq signal presented in the supplementary table [18] was used, and the nucleosomes that were detected closest to the location of the ChEC-seq signal were targeted for calculating fuzziness using Danpos2 [19]. **b**, **c** Paused genes (with the enriched H3K4me3) whose nucleosome fuzziness was increased upon the loss of Ino80p (N = 1,470) were binned into quartiles based on the changes in nucleosome fuzziness, where Q4 represents the highest changes. Average profiles of median MNase-seq intensity in the Ctrl and KD samples (**b**) or Ino80p ChEC-seq intensity (**c**) at each quartile.

Discussion

**Reviewer’s comment:**

33. First line. I don't agree that the way the data are discussed indicates things are same between yeast and human- because discussion is focused on pausing at 5' end not the potential obstacle of +1 nucleosome, which is detected by altered distribution of PRO-seq reads from existing pauses that are based on different mechanisms toward new positions that may reflect inability to transit an Ino80-sensitive barrier.

**Our response:**

In this part, we would like to discuss the common features of RNAPⅡ pausing at the protein-coding genes, not the Ino80p-dependent shift-to-5’ genes. We would like to argue that the non-uniform distribution pattern that we observed in PRO-seq data was reminiscent of promoter-proximal pausing in metazoans. We revised the text to make it more clearly what we tried to convey in the Discussion (p20-21) section.

**Reviewer’s comment:**

34. p 17 line 22-26 "One of the reasons for this discrepancy might be that NELF, which is involved in the pausing of RNAPII at a more promoter-proximal region [54], is not conserved in yeasts, including S. cerevisiae and S. pombe [55, 56].

Both NELF and negative activity of DSIF. These ideas are already widely suggested in the literature so should be discussed differently.

**Our comment:**

We agree that the previous study we cited [20] showed that the knockdown of DSIF also led to a downstream shift of pause location. This result could be expected in that NELF and DSIF cooperatively interact with the elongation complex [21]. We additionally cited the literature to imply that the cooperative binding of both factors is involved in the RNAPⅡ pausing at a more promoter-proximal region.

35. p17 line 28 "The hypothesis is consistent with the PRO-seq distribution reported for S. pombe, which showed a near-overlapping association of RNAPII pausing with the +1 dyad [17]."

**Reviewer’s comment:**

36. p17 line 34 language is awkward.

**Our response:**

We revised the manuscript more precisely.

**Reviewer’s comment:**

37. p17 line 37. There is no basis for "surprisingly" as mechanisms of pausing are different.

**Our response:**

We agree that a distinct phenomenon in two different organisms could be probable. We deleted the word “surprisingly”.

**Reviewer’s comment:**

38. p18 line 28-35. An alternative is that Ino80 enables elongation through the P2 and is defective. It is difficult to say that relative reduction of P1 is happening or if an increase in P2 is from other populations that are releasing from P1 normally, either into what should be productive elongation or promoter proximal termination.

**Our response:**

We agree that PRO-seq data alone could not distinguish whether the RNAPII population at P1 is interconnected to those at P2 or not. The RNAPII population at P1 could not normally release to P2 upon the loss of INO80 and could be subject to premature termination. Thus, we deleted the phrase “probably allows further elongation of RNAPII” and added the mentioned possibility at the end of the paragraph in the Discussion (p22) sections.

Conclusion

**Reviewer’s comment:**

39. Reconsider reframing based on points above.

**Our response:**

We have thoroughly revised the entire manuscript according to all the reviewer’s comments, including several suggestions, queries, and concerns.

**Reviewer’s comment:**

40. Please look over all figures for ways to improve figure labeling and figure legends, i.e. labeling of dashed lines on figures, annotation of datasets, schematics for metrics, etc.

**Our response:**

As discussed above (reviewer 2’s general points 1 and 3), we revised the figures to be more accessible by changing labeling more precisely or adding the labeling to previously unlabeled one, such as the dashed lines on figures. We also added the description of schematics for metrics in more detail.

P.S. The following is a detailed list of changes made to the revised manuscript:

1) In order to respond to the additional issues 3, 4, 6 and 7 of Reviewer 1 and the other points 15, 16, and 37 of Reviewer 2, we thoroughly revised the relevant part in the Results and the Discussion section. In doing so, we edited the typo and changed word choice.
2) In order to respond to the additional issues 1 and 2 of Reviewer 1 and the other points 11, 12, 17, 20, 23, 24, 27, 33, and 36 of Reviewer 2, we added the description about not only the data and speculation but also the calculation metric in more detail in the relevant Results section so that it would be more readily understandable.
3) In order to respond to the general point 1 of Reviewer 2, we examined the positive correlation between the published Rbp3 NET-seq and ChIP-exo data within both promoter-proximal and gene body regions (Fig. S5d). All positive correlations between PRO-seq, NET-seq, and ChIP-exo helped us reconcile the general distribution pattern of elongating RNAPⅡ across genes detected by several different techniques in *S. cerevisiae*.
4) In order to respond to the general point 2 of Reviewer 2, we added the description of the main drawbacks in the PRO-seq technique in the Introduction (p3, 5) section. We also stated the precise explanation about the difference between the experimental methods used in our study and the previous study that we mainly compared [5] in the Results (p7-8) section.
5) In order to respond to the general point 3 and the other point 40 of Reviewer 2, we labeled the figures more readily understandable. Particularly, it was briefly indicated what the sample was below or above the sample accession number (GSM) (Fig. 1, S1-3), or figures were simply labeled by the indicated methods or samples. The detailed information of the sample accession number was described in the legend. We also added the labeling to the previously unlabeled one, including the dashed lines on figures (e.g., the 10^th^ and 90^th^ percentiles of the relative position of P2 with respect to P1; Fig. 3d, 4a, b, 5c, e).
6) In order to respond to the other points 4 and 9 of Reviewer 2, we added the description in the Abstract (p2) and the Introduction (p5-6) sections so that it would be easier to understand the difference between the main and the alternative pausing site.
7) In order to respond to the other points 5, 10, 28, and 29 of Reviewer 2, we revised the manuscript in the Abstract (p2), Introduction (p6), and Results (p18-19, 20) section. In doing so, we proposed our hypothesis about the role of the INO80 to mediate the RNAPⅡ pausing site determination, which is highly associated with the +1 nucleosome, in a tone-downed state.
8) In order to respond to the other points 6, 7, 8, 18, and 34 of Reviewer 2, we revised the Introduction, Results, and Discussion sections to be more sophisticated. In doing so, we included substantially more literature in the References. Particularly, we added the referencing the previous literature suggesting the conserved pausing-like features in *S. cerevisiae* [22] and those about H2A.Z dimer exchange activity of Ino80p [9-12] in the Introduction (p3, 5) sections. We further referenced the previous study about the cooperative interaction between NELF and DSIF to RNAPⅡ in the Discussion (p21) sections. We also deleted the incorrectly cited [13] and revised the manuscript to state that the role of Ino80p in transcriptional regulation, not elongation, was evident in the Results (p12) sections. Furthermore, we added the description of the major drawbacks of PRO-seq experiments in the Introduction (p3, 5) sections.
9) In order to respond to the other point 12 of Reviewer 2, we analyzed the scatter plot of the PI between the previously published PRO-seq data set and our data set (Fig. 1c, S1b, S2b, S3b).
10) In order to respond to the other point 13 of Reviewer 2, we generated the average profiles of median PRO-seq intensity for the genes that were grouped into the quartiles based on the PI (Fig. 1d, e, S2c, d, S3c, d).
11) In order to respond to the other point 14 of Reviewer 2, we visualized the boxplot of the PI of *wt* and *spt4*Δ samples in the previously published data set or this study (Fig. S1c, d).
12) In order to respond to the other point 22 of Reviewer 2, we analyzed the boxplot of retained ratio at P1 of no-shift and shifted genes in both *S. cerevisiae* (Fig. S8d) and mESCs (Fig. S10f).
13) In order to respond to the other point 30 of Reviewer 2, we generated the average profile of median MNase-seq of 1,000 bootstrap samples around P1 in *S. cerevisiae* (Fig. 4a) and mESCs (Fig. 5e).
14) In addition, we visualized the distribution pattern of the PRO-cap signal at the observed TSS (Fig. S4a) and generated the sequence preference (Fig. S4b) that was markedly similar to the previously reported [5, 23]. Furthermore, we recognized the mistake of using the paired t-test when conducting the empirical statistical test of the *arp5*Δ set, and corrected it to use a t-test, and proceeded again. We found strikingly similar results that the deletion of Arp5p caused the significant increase of upstream peaks (Fig. 4e-f, S9d-g) as in the previous manuscript.

**References**

1.	Beckwith SL, Schwartz EK, García-Nieto PE, King DA, Gowans GJ, Wong KM, Eckley TL, Paraschuk AP, Peltan EL, Lee LR, et al: **The INO80 chromatin remodeler sustains metabolic stability by promoting TOR signaling and regulating histone acetylation.** *PLoS Genet* 2018, **14:**e1007216.

2.	Sanz LA, Hartono SR, Lim YW, Steyaert S, Rajpurkar A, Ginno PA, Xu X, Chédin F: **Prevalent, Dynamic, and Conserved R-Loop Structures Associate with Specific Epigenomic Signatures in Mammals.** *Mol Cell* 2016, **63:**167-178.

3.	Wahba L, Costantino L, Tan FJ, Zimmer A, Koshland D: **S1-DRIP-seq identifies high expression and polyA tracts as major contributors to R-loop formation.** *Genes Dev* 2016, **30:**1327-1338.

4.	Feldman JL, Peterson CL: **Yeast Sirtuin Family Members Maintain Transcription Homeostasis to Ensure Genome Stability.** *Cell Rep* 2019, **27:**2978-2989.e2975.

5.	Booth GT, Wang IX, Cheung VG, Lis JT: **Divergence of a conserved elongation factor and transcription regulation in budding and fission yeast.** *Genome Res* 2016, **26:**799-811.

6.	Core LJ, Waterfall JJ, Lis JT: **Nascent RNA sequencing reveals widespread pausing and divergent initiation at human promoters.** *Science* 2008, **322:**1845-1848.

7.	Mahat DB, Kwak H, Booth GT, Jonkers IH, Danko CG, Patel RK, Waters CT, Munson K, Core LJ, Lis JT: **Base-pair-resolution genome-wide mapping of active RNA polymerases using precision nuclear run-on (PRO-seq).** *Nat Protoc* 2016, **11:**1455-1476.

8.	Kwak H, Fuda NJ, Core LJ, Lis JT: **Precise maps of RNA polymerase reveal how promoters direct initiation and pausing.** *Science* 2013, **339:**950-953.

9.	Wang F, Ranjan A, Wei D, Wu C: **Comment on "A histone acetylation switch regulates H2A.Z deposition by the SWR-C remodeling enzyme".** *Science* 2016, **353:**358.

10.	Luk E, Ranjan A, Fitzgerald PC, Mizuguchi G, Huang Y, Wei D, Wu C: **Stepwise histone replacement by SWR1 requires dual activation with histone H2A.Z and canonical nucleosome.** *Cell* 2010, **143:**725-736.

11.	Watanabe S, Peterson CL: **Response to Comment on "A histone acetylation switch regulates H2A.Z deposition by the SWR-C remodeling enzyme".** *Science* 2016, **353:**358.

12.	Watanabe S, Radman-Livaja M, Rando OJ, Peterson CL: **A histone acetylation switch regulates H2A.Z deposition by the SWR-C remodeling enzyme.** *Science* 2013, **340:**195-199.

13.	Poli J, Gerhold CB, Tosi A, Hustedt N, Seeber A, Sack R, Herzog F, Pasero P, Shimada K, Hopfner KP, Gasser SM: **Mec1, INO80, and the PAF1 complex cooperate to limit transcription replication conflicts through RNAPII removal during replication stress.** *Genes Dev* 2016, **30:**337-354.

14.	Lafon A, Taranum S, Pietrocola F, Dingli F, Loew D, Brahma S, Bartholomew B, Papamichos-Chronakis M: **INO80 Chromatin Remodeler Facilitates Release of RNA Polymerase II from Chromatin for Ubiquitin-Mediated Proteasomal Degradation.** *Mol Cell* 2015, **60:**784-796.

15.	Booth GT, Parua PK, Sansó M, Fisher RP, Lis JT: **Cdk9 regulates a promoter-proximal checkpoint to modulate RNA polymerase II elongation rate in fission yeast.** *Nat Commun* 2018, **9:**543.

16.	Rossi MJ, Kuntala PK, Lai WK, Yamada N, Badjatia N, Mittal C, Kuzu G, Bocklund K, Farrell NP, Blanda TR: **A high-resolution protein architecture of the budding yeast genome.** *Nature* 2021, **592:**309-314.

17.	Wang L, Du Y, Ward JM, Shimbo T, Lackford B, Zheng X, Miao YL, Zhou B, Han L, Fargo DC, et al: **INO80 facilitates pluripotency gene activation in embryonic stem cell self-renewal, reprogramming, and blastocyst development.** *Cell Stem Cell* 2014, **14:**575-591.

18.	Kubik S, Bruzzone MJ, Challal D, Dreos R, Mattarocci S, Bucher P, Libri D, Shore D: **Opposing chromatin remodelers control transcription initiation frequency and start site selection.** *Nat Struct Mol Biol* 2019, **26:**744-754.

19.	Chen K, Xi Y, Pan X, Li Z, Kaestner K, Tyler J, Dent S, He X, Li W: **DANPOS: dynamic analysis of nucleosome position and occupancy by sequencing.** *Genome Res* 2013, **23:**341-351.

20.	Li J, Liu Y, Rhee HS, Ghosh SK, Bai L, Pugh BF, Gilmour DS: **Kinetic competition between elongation rate and binding of NELF controls promoter-proximal pausing.** *Mol Cell* 2013, **50:**711-722.

21.	Missra A, Gilmour DS: **Interactions between DSIF (DRB sensitivity inducing factor), NELF (negative elongation factor), and the Drosophila RNA polymerase II transcription elongation complex.** *Proc Natl Acad Sci U S A* 2010, **107:**11301-11306.

22.	Venters BJ, Pugh BF: **A canonical promoter organization of the transcription machinery and its regulators in the Saccharomyces genome.** *Genome Res* 2009, **19:**360-371.

23.	Doris SM, Chuang J, Viktorovskaya O, Murawska M, Spatt D, Churchman LS, Winston F: **Spt6 Is Required for the Fidelity of Promoter Selection.** *Mol Cell* 2018, **72:**687-699.e686.

**2^nd^ round**

**Reviewer 1**

The authors carefully and robustly addressed the reviewers' comments.

**Reviewer 2**

My major comments to the authors are to attempt to make the work as accessible as possible while at the same time showing awareness of the literature, alternative models/interpretations, and clarity of language to guide readers through the manuscript. Their extensive rebuttal is appreciated. However, rebuttal to reviewers that includes negative results in response to obvious questions does a disservice to readers that might have the same obvious questions and these are not addressed in the manuscript proper. Furthermore, the authors do include indication of data reproducibility in a table, but this is not at the appropriate level. If authors use many different metrics, including those at single base resolution, some demonstration of reproducibility at that level would be welcome and careful. From examination of curves that authors do provide, it does appear that the data are quite reproducible. It is the authors' responsibility to make sure the readers clearly appreciate this.

For the authors' consideration: one feature in common for both yeast and human promoters that show Ino80 effects- these promoters have more highly phased nucleosomes and higher occupancy (Figure 4A, Fig 5E). Again, this similarity that +1 nucleosome has different attributes for the promoters affected, and that there is increase in +1 nucleosome overlying RNAP II in both cases (S. cerevisiae shifting from downstream of +1 into the +1, and potentially either release of RNAP II from pause site in humans OR an increase in amount of RNAP II that normally elongates through the +1 after pause release being captured). It seems like this might be much more clearly stated.

I have provided the rest of my comments in the form of an annotated PDF of the manuscript.

**Authors’ response**

Reviewer #2: Cheon et al present a revised manuscript titled "The chromatin remodeler Ino80 mediates RNAPⅡ pausing site determination"

My major comments to the authors are to attempt to make the work as accessible as possible while at the same time showing awareness of the literature, alternative models/interpretations, and clarity of language to guide readers through the manuscript. Their extensive rebuttal is appreciated.

**Our response:**

We thank the reviewer for the positive evaluation of our work.

**Reviewer’s comment:**

However, rebuttal to reviewers that includes negative results in response to obvious questions does a disservice to readers that might have the same obvious questions and these are not addressed in the manuscript proper.

**Our response:**

We added the relevant part in the Discussion (p22-23) section. We are also eager to open our rebuttal works to the public if our manuscript is published in Genome Biology.

**Reviewer’s comment:**

Furthermore, the authors do include indication of data reproducibility in a table, but this is not at the appropriate level. If authors use many different metrics, including those at single base resolution, some demonstration of reproducibility at that level would be welcome and careful. From examination of curves that authors do provide, it does appear that the data are quite reproducible. It is the authors' responsibility to make sure the readers clearly appreciate this.

**Our response:**

Thank you for the suggestion. We agree with the importance of reproducibility when analyzing the data generated using biological replicates. However, we would like to note that Fig. 3a showed the reproducible transition of the average curve in Ino80p depletion at each replicate. We also determined all the pausing sites (P1 and P2), considering the variance of replicates. Furthermore, the changes upon the Ino80p-KD showed the tendency to counteract in the subsequent rescue conditions at each replicate (Fig. 3b), even we did not consider the rescue samples when identifying P2. Thus, we believe that our PRO-seq data is reproducible and that the pausing site identifying method worked as well to analyze our PRO-seq data considering each replicate of two samples, Ctrl and KD.

**Reviewer’s comment:**

For the authors' consideration: one feature in common for both yeast and human promoters that show Ino80 effects- these promoters have more highly phased nucleosomes and higher occupancy (Figure 4A, Fig 5E). Again, this similarity that +1 nucleosome has different attributes for the promoters affected, and that there is increase in +1 nucleosome overlying RNAP II in both cases (S. cerevisiae shifting from downstream of +1 into the +1, and potentially either release of RNAP II from pause site in humans OR an increase in amount of RNAP II that normally elongates through the +1 after pause release being captured). It seems like this might be much more clearly stated.

**Our response:**

Thank you for the valuable comments. We added the relevant part in the last paragraph of the Discussion (p22-23) section and modified the Conclusion (p23) section. We tone-downed and rewrote the manuscript so that our conclusions are not overstated, in particular, both the extent of the similarities between RNAPⅡ phenotype of *S. cerevisiae* and mESCs and the interpretation of RNAPⅡ pausing at the alternative pausing site in Ino80p depletion.

I have provided the rest of my comments in the form of an annotated PDF of the manuscript.Reviewer #2:

**Our response:**

We corrected our manuscript according to the reviewer’s comments.

In particular, the respond to the comment about Fig. 5g (‘I am truly sorry but I still do not understand this. Genes were identified by a shift in distribution- how will the shift in distribution not generally result in a reduction of P1?’) is as follows: We are sorry to misunderstand your previous comment in the first-round review. The difference between Fig. 5d and 5g was that Fig. 5d was generated considering only the reads themselves at the indicated pausing sites. Instead, Fig. 5g was generated by ‘the reads at the indicated pausing sites / summation of reads at all the identified peaks within PR regions’ (‘/’ means division). We intended to argue that the amount of RNAPⅡ at the specific site is indeed changed even when considering the total PR RNAPⅡ. However, we also agree with that these analyses are recursive in part so that we moved the related data to the supplementary figures (Fig. S9c, g, and Fig. S10h).
